# iTACTIC – implementing Treatment Algorithms for the Correction of Trauma-Induced Coagulopathy: study protocol for a multicentre, randomised controlled trial

**DOI:** 10.1186/s13063-017-2224-9

**Published:** 2017-10-18

**Authors:** Kjersti Baksaas-Aasen, Lewis Gall, Simon Eaglestone, Claire Rourke, Nicole. P. Juffermans, J. Carel Goslings, Paal Aksel Naess, Susan van Dieren, Sisse Rye Ostrowski, Jakob Stensballe, Marc Maegele, Simon J. Stanworth, Christine Gaarder, Karim Brohi, Per I. Johansson

**Affiliations:** 1 Department of Traumatology, Oslo University Hospital, Oslo, Norway; 2 Centre for Trauma Sciences, Blizard Institute, Queen Mary University of London, London, UK; 3 Department of Intensive Care Medicine, Academic Medical Center, Amsterdam, The Netherlands; 4 Trauma Unit, Department of Surgery, Academic Medical Center, Amsterdam, The Netherlands; 5 Department for Traumatology and Orthopedic Surgery, Cologne-Merheim Medical Centre, University of Witten/Herdecke, Cologne, Germany; 6 NHS Blood and Transplant/Oxford University Hospital NHS Trust, John Radcliffe Hospital, Oxford, UK; 7 Radcliffe Department of Medicine, University of Oxford, Oxford, UK; 8 Section for Transfusion Medicine, Capital Region Blood Bank, Copenhagen University Hospital, Rigshospitalet, Copenhagen, Denmark

**Keywords:** Trauma, Haemorrhage, Trauma-induced coagulopathy, Viscoelastic haemostatic assays, Randomised control trial, Conventional coagulation tests, Transfusion

## Abstract

**Background:**

Traumatic injury is the fourth leading cause of death globally. Half of all trauma deaths are due to bleeding and most of these will occur within 6 h of injury. Haemorrhagic shock following injury has been shown to induce a clotting dysfunction within minutes, and this early trauma-induced coagulopathy (TIC) may exacerbate bleeding and is associated with higher mortality and morbidity. In spite of improved resuscitation strategies over the last decade, current transfusion therapy still fails to correct TIC during ongoing haemorrhage and evidence for the optimal management of bleeding trauma patients is lacking. Recent publications describe increasing the use of Viscoelastic Haemostatic Assays (VHAs) in trauma haemorrhage; however, there is insufficient evidence to support their superiority to conventional coagulation tests (CCTs).

**Methods/design:**

This multicentre, randomised controlled study will compare the haemostatic effect of an evidence-based VHA-guided versus an optimised CCT-guided transfusion algorithm in haemorrhaging trauma patients. A total of 392 adult trauma patients will be enrolled at major trauma centres. Participants will be eligible if they present with clinical signs of haemorrhagic shock, activate the local massive haemorrhage protocol and initiate first blood transfusion. Enrolled patients will be block randomised per centre to either VHA-guided or CCT-guided transfusion therapy in addition to that therapy delivered as part of standard care, until haemostasis is achieved. Patients will be followed until discharge or 28 days. The primary endpoint is the proportion of subjects alive and free of massive transfusion (less than 10 units of red blood cells) at 24 h. Secondary outcomes include the effect of CCT- versus VHA-guided therapy on organ failure, total hospital and intensive care lengths of stay, health care resources needed and mortality. Surviving patients will be asked to complete a quality of life questionnaire (EuroQol EQ-5D^TM^) at day 90.

**Discussion:**

CCTs have traditionally been used to detect TIC and monitor response to treatment in traumatic major haemorrhage. The use of VHAs is increasing, but limited evidence exists to support the superiority of these technologies (or comparatively) for patient-centred outcomes. This knowledge gap will be addressed by this trial.

**Trial registration:**

ClinicalTrials.gov, ID: NCT02593877. Registered on 15 October 2015.

**Trial sponsor**

Queen Mary University of London

The contact person of the above sponsor organisation is: Dr. Sally Burtles, Director of Research Services and Business Development, Joint Research Management Office, QM Innovation Building, 5 Walden Street, London E1 2EF; phone: 020 7882 7260; Email: sponsorsrep@bartshealth.nhs.uk

**Trial sites**

Academic Medical Centre, Amsterdam, The Netherlands

Kliniken der Stadt Köln gGmbH, Cologne, Germany

Rigshospitalet (Copenhagen University Hospital), Copenhagen, Denmark

John Radcliff Hospital, Oxford, United Kingdom

Oslo University Hospital, Oslo, Norway

The Royal London Hospital, London, United Kingdom

Centre for Trauma Sciences, Blizard Institute, Queen Mary University of London, London, United Kingdom

Health Economics Research Centre, Nuffield Department of Population Health, University of Oxford, Oxford, United Kingdom

**Sites that are planning to start recruitment in mid/late 2017**

Nottingham University Hospitals, Queen’s Medical Centre, Nottingham, United Kingdom

University of Kansas Hospital (UKH), Kansas City, MO, USA

**Protocol version**: 3.0/14.03.2017 (Additional file 1)

**Electronic supplementary material:**

The online version of this article (doi:10.1186/s13063-017-2224-9) contains supplementary material, which is available to authorized users.

## Background

Traumatic injury is responsible for a large and increasing proportion of the world’s burden of disease and is the fourth leading cause of death globally [1]. Half of all trauma deaths are due to bleeding and most of these will occur within 6 h from injury [2]. Haemorrhagic shock following injury has been shown to induce a clotting dysfunction (i.e. coagulopathy) within minutes [3–5]. Such early trauma-induced coagulopathy (TIC) may exacerbate bleeding and is associated with higher mortality and morbidity [4, 6, 7]. Many more injured patients will go on to develop different types of coagulopathy at different times during the course of their treatment, either as a result of their body’s ongoing response to trauma or as a consequence of their clinical care. Coagulopathic, haemorrhaging trauma patients have increased blood transfusion requirements, increased mortality and more adverse outcomes [8]. Despite improvements in surgical techniques, resuscitation strategies and intensive care treatments, outcomes for critically injured patients remain poor [9]. Within the last decade research focussing on TIC has led to improved resuscitation strategies, resulting in the early and more aggressive use of blood products and coagulation factors for the management of massively bleeding patients.

In spite of improved resuscitation strategies, current transfusion therapy still fails to correct coagulopathy during ongoing haemorrhage [10, 11]. The mechanisms and genesis of TIC have yet to be fully elucidated, and there are many questions about how to optimally diagnose, resuscitate and monitor the critically bleeding trauma patient. It is important to detect TIC as early as possible. Conventional coagulation tests (CCT), such as prothrombin time/international normalised ratio (PT/INR), activated partial thromboplastin time (APTT), fibrinogen concentration and PLT, have traditionally been used. However, there is a striking lack of evidence to support the use of these CCTs to monitor resuscitation, although threshold triggers for intervention based on CCTs have been suggested [5]. Recent published evidence describes an increasing recognition for the potential of the two current market-leading Viscoelastic Haemostatic Assays (VHAs) namely thromboelastography (TEG®; Haemonetics Incorporation) and rotational thromboelastometry (ROTEM®; TEM Innovation GmbH). Both platforms use similar test modes to rapidly and accurately determine the functional coagulation status of patient whole blood. However, the evidence base supporting a role for these VHA devices is limited, and less attention has been directed to understanding their cost-effectiveness. Cost-effectiveness may be particularly relevant both in the context of additional therapeutic interventions required, but also in potential savings, if fewer treatments are required based on delivery of individualised assessments of haemostasis.

The relative contribution of blood components, such as fibrinogen and platelets, to clot strength can be evaluated through the use of specific inhibitors or agonists [12]. The viscoelastic properties of blood samples are recorded under low shear conditions, thereby providing a comprehensive visual profile of clot formation and breakdown (fibrinolysis).

Unlike laboratory-based CCTs which might take more than 60 min for the results to be available to clinicians [8], VHA is a point-of-care device which might provide clinically relevant results within even 5–10 min and thus may be repeated in a massive bleeding situation to identify patient-specific needs for transfusion components in a timelier manner. Furthermore, VHAs provide the potential to detect hyperfibrinolysis, and possibly hypercoagulability. However, VHA assays and testing have costs, require training and additional oversight, and may not provide insight into other potentially important haemostatic derangements at the endothelial or platelet level. In addition, other publications attest to how changes in the process and pathways for the delivery of CCTs can be modified and accelerated [13, 14].

Whilst VHA has been used for many years in liver transplant and cardio-pulmonary surgery, robust data supporting its universal uptake in the context of trauma are lacking. Whilst some publications have attempted to identify VHA patterns and thresholds characterizing TIC and the need for massive transfusion in trauma patients, definitive evidence proving its superiority over CCTs in the diagnosis and management of coagulopathy in the acute setting is not available [15–18].

Although considered a preventable major cause of death, the management of coagulopathic bleeding in trauma patients remains primarily based upon retrospective registry studies of survival and extrapolating the results of transfusion practice performed in the elective, non-acute surgical setting. Treatment is diverse comprising the empiric transfusion of red blood cells (RBC) and clotting product supplements to patients, blind to the type and severity of TIC they may have – or indeed even if they do not have coagulopathy. It is well established that blood transfusion carries significant health risks both related to transmission of pathogens and to the development of transfusion reactions. Published in 2015, the results of the Pragmatic, Randomised Optimal Platelet and Plasma Ratios (PROPPR) trial [19] provide the best evidence to date for optimal trauma haemorrhage resuscitation. PROPPR demonstrated that an empiric massive transfusion protocol (MTP) aiming at a ratio of 1:1:1 of blood components (RBC 1: plasma 1: platelets 1) administered from the early phase of care and during ongoing haemorrhage was associated with fewer exsanguinations in the initial 24 h (*p* = 0.03) and a tendency towards improved 24-h survival (*p* = 0.12) than a 1:1:2 ratio.

The present prospective randomised controlled trial (RCT) will employ evidence-based treatment algorithms to compare outcomes of VHA-guided resuscitation versus CCT resuscitation support in haemorrhaging trauma patients.

The hypothesis for this comparative study is that VHA-directed therapy will enhance early haemostatic control by the targeted correction of TIC, whilst also reducing the total amount of blood products and procoagulants administered to all bleeding trauma patients, including those not having TIC. This would significantly reduce both the number of patients receiving blood transfusion and the number of transfused blood products per transfused patient, thereby improving both patient safety and resource utilisation.

## Methods/design

This is an investigator-initiated, multi-centred, superiority, parallel-group, randomised controlled trial performed at eight major trauma centres. The trial sites include: Rigshospitalet (Copenhagen, Denmark), Academic Medical Centre (Amsterdam, The Netherlands), Oslo University Hospital (Oslo, Norway), Kliniken der Stadt Köln gGmbH Cologne, Germany), The Royal London Hospital (London, UK), John Radcliffe Hospital (Oxford, UK). Nottingham University Hospitals, Queen’s Medical Centre (Nottingham, United Kingdom) and University of Kansas Hospital (UKH) (Kansas City, MO, USA) are planning to start recruitment in 2017.

**Fig. 1:**
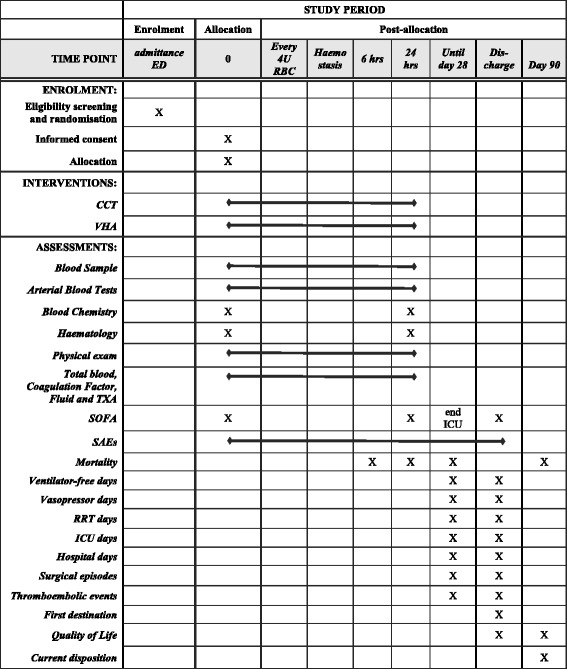
Standard Protocol Items: Recommendation for Interventional Trials (SPIRIT) schedule of enrolment, interventions and assessments

This protocol (Additional file 1) conforms to the Consolidated Standard of Reporting Trials (CONSORT) guidelines. Figure 1 shows the Standard Protocol Items: Recommendation for Interventional Trials (SPIRIT) schedule of enrolment, interventions and assessments. The SPIRIT Checklist is given in the Additional file 2.

**Fig. 2:**
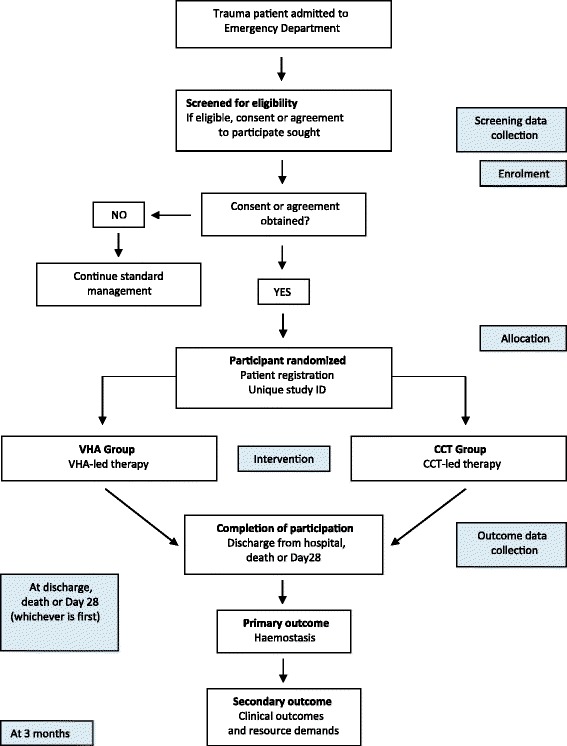
Study scheme

An overview of the study process is provided in Fig. 2 (study scheme).

### Inclusion criteria

Adult trauma patients (according to local definitions) will be enrolled if they present with clinical signs of haemorrhagic shock, according to the responsible trauma team leader, activate the local massive haemorrhage protocol, according to the participating institutions’ specific routines, and initiate first transfusion. Participants must be randomised within 3 h of injury and 1 h of admission to the ED of the participating study site. Agreement is provided on behalf of incapacitated patients by a personal consultee (PC) or a nominated consultee (NC).

### Exclusion criteria

There are no exclusion criteria.

### Primary objective

The primary objective is to compare the haemostatic effect of VHA assay-guided transfusion strategy versus optimised CCT-guided transfusion strategy in haemorrhaging trauma patients.

### Secondary objective

The secondary objectives of the study are to determine the effects of VHA-led versus optimised CCT-guided resuscitation on organ failure, hospital length of stay (LOS), intensive care unit (ICU) stay, duration of mechanical ventilation, health care resource needs and mortality.

### Primary endpoint

The primary endpoint is the proportion of subjects alive and free of massive transfusion (less than 10 units of RBC transfused) at 24 h post admission.

### Secondary endpoints

The secondary endpoints listed below will be analysed in order to provide a sensitive and comprehensive description of outcomes and health care resource demands for the VHA and CCT arm subjects:

- All-cause mortality at 6 and 24 h and 28 and 90 days post admission
- Duration and severity of coagulopathy until haemostasis, as defined by the area under the time multiplied by Prothrombin Ratio (PTr) curve, with coagulopathy defined as PTr > 1.2. Patients who die will have their time of haemostasis set at 24 h, and last PTr extrapolated to this time point
- Proportion of patients who have corrected coagulopathy, defined as PTr < 1.2, after first 8 units of RBC
- Time to haemostasis (defined as having occurred at the end of the first hour free of red cell transfusions and the treating clinicians believe primary haemostasis has been achieved)
- Time spent in coagulopathic condition, defined as PTr ≥ 1.2, until haemostasis, defined as the point 1 h from the last administration of RBC and the treating clinician believes that primary haemostasis has been achieved
- Blood products administered (RBC, plasma, platelets alone and in total) within first 6 and 24 h after admission
- 28-day ventilator-free and ICU-free days (patients who die in hospital during the 28-day study period will be considered to have zero hospital-free days)
- Total hospital length of stay
- 28-day symptomatic thromboembolic events defined as: deep venous thrombosis (DVT) diagnosed by compression ultrasound or venography, pulmonary embolism diagnosed by computed tomography (CT) pulmonary angiogram or ventilation-perfusion scan or myocardial infarction and/or stroke identified by standard clinical diagnostic investigation(s)
- Incidence of transfusion-related complications
- Incidence of organ dysfunction, defined by Sequential Organ Failure Assessment (SOFA) score
- Health care resource, productivity costs and HRQoL (EuroQol EQ-5DTM at discharge or day 28, and at day 90)
- Lifetime health economic cost-effectiveness of personalised VHA-guided haemorrhagic treatment versus MTP-based on best practice and CCTs

### Study procedures

#### Screening procedures

Local investigators will identify eligible adult trauma patients with haemorrhagic shock and ongoing bleeding as soon as possible after the patient has arrived in the emergency department (ED), using local transfusion triggers. If patients are deemed to be eligible, consent for entry into the trial will be sought. A screening log will be completed once a week, which will record all patients considered for eligibility to the trial by the investigator(s). The log will include age, gender, inclusion/exclusion criteria and other reasons for non-enrolment. The screening log data will be reviewed at regular intervals.

#### Randomisation procedure

Enrolled patients will be block randomised per centre to either the CCT or the VHA study arm within 3 h of injury and within 1 h of admission. The trial will be un-blinded for clinical staff and site investigators. Once a patient is determined eligible for the study and informed consent or agreement has been obtained, each subject will be enrolled as soon as possible and will be assigned a unique alphanumeric study identifier. Randomisation will be performed by the site investigator opening a sealed envelope containing the randomised treatment group, to allow for immediate allocation of subjects. An independent party, appointed by the sponsor, will generate the randomisation sequence and site envelopes centrally.

#### Schedule of intervention

All participating centres will initiate the management of the study population according to standard local protocols regardless of enrolment in the trial. Following randomisation, study participants will undergo interventions at set time points as outlined in Fig. 1 (Standard Protocol Items: Recommendations for Interventional Trials (SPIRIT) schedule of enrolment, interventions and assessments) and be followed up for 90 days after enrolment.

### Standard care

All participating centres currently manage critically bleeding trauma patients according to a standardised MTP aiming at a ratio of RBC 1: plasma 1: platelets 1 (1:1:1), typically administering plasma from the start of resuscitation and platelets immediately as they become available. Tranexamic acid (TXA) will be administered to all patients as an intravenous bolus of 1 g over 10 min (either pre-hospital or in the ED) followed by an intravenous infusion of 1 g over 8 h, providing that the patient is less than 3 h post injury.

Current use of additional diagnostics and therapy, such as systematic approach according to Advanced Trauma Life Support (ATLS®) principles, early imaging (e.g. X-rays, Focussed Assessment with Sonography for Trauma (FAST), computer tomography (CT)), activation criteria for MTP, surgical approach applying damage control principles when indicated, the availability and use of interventional radiology, will not be affected in either of the study groups. An optimised initial MTP based on a 1:1:1 balanced transfusion will be implemented in all centres for approximately 2 months prior to initiation of the RCT and standardised as far as local routines and blood product availability allow.

### Initiation of study care

**Fig. 3:**
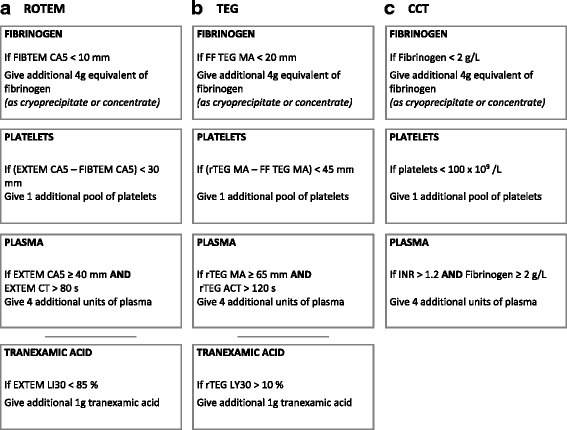
Algorithms

Corresponding and optimised algorithms based on VHA trigger parameters for the VHA arm and CCT results for the CCT arm, respectively, have been developed and will be applied in the enrolled subjects (Fig. 3a, b, c). During active haemorrhage, samples will be taken for CCT/VHA analysis at baseline and after every 4 units of RBC until haemostasis. The results from each blood sample will be acted upon as soon as they are available. For the VHA arm, this implies acting upon the parameters as they are appearing, not waiting until the VHA trace is completed.

If a planned study intervention has not yet been administered, the sample will be taken and analysed (where resources allow) but will not be used to guide study intervention. The first sample taken after a study intervention is actually administered will be the next sample used to guide study intervention based upon the respective protocol.

The same blood products and procoagulants will be employed in both study arms, with existing standard practice in all participating centres being closely aligned to that of the CCT arm. Trial products will be given as an addition to the 1:1:1 baseline MTP and TXA. The procoagulants included in the algorithms are fibrinogen concentrate or cryoprecipitate and additional platelets, plasma and TXA.

Enrolled patients will be block randomised per centre to either study arm:

- CCT: haemostatic resuscitation (standard care), based on a MTP aiming at ratio 1:1:1 of blood components (RBC 1: plasma 1: platelets 1) and TXA, and CCTs to guide further resuscitation with blood products and procoagulant factors
- VHA: haemostatic resuscitation (standard care), based on a MTP aiming at ratio 1:1:1 of blood components (RBC 1: plasma 1: platelets 1) and TXA, and VHA-guiding further resuscitation with blood products and procoagulant factors

### Randomised study care – VHA arm

VHA will be conducted for all subjects in the VHA arm at each time point up to 24 h. During active haemorrhage, samples will be taken for VHA analysis at baseline and after every 4 units of RBC until haemostasis. The clinical course of subjects randomised to the VHA arm will follow a treatment algorithm utilizing VHA results (Fig. 3a and b). The subject will be treated with standard haemostatic resuscitation based on a MTP aiming for 1:1:1 ratio of blood components and initial TXA. In addition, according to threshold values in the algorithms, the subjects will be given fibrinogen concentrate or cryoprecipitate and/or additional platelets, plasma and TXA depending on the VHA results.

Fibrinogen concentrate or cryoprecipitate will be administrated when FIBTEM Clot Amplitude at 5 min (CA5) is below 10 mm or functional fibrinogen (FF) TEG® Maximum Amplitude (MA) is below 20 mm. Additional platelets are indicated when the subtracted amplitude of FIBTEM CA5 from EXTEM CA5 is below 30 mm, or the subtracted amplitude of FF TEG® MA from rapid TEG® MA is below 45 mm. Additional plasma is indicated when the results show a normal EXTEM CA5, defined as above 40 mm, but still a prolonged EXTEM clotting time (CT), defined as above 80 s, or normal rapid TEG® MA, defined as above 65 mm, but still a prolonged rapid TEG® activated clotting time (ACT), defined as above 120 s. Additional TXA is indicated when the EXTEM Lysis Index at 30 min (LI30) is below 85% or rapid TEG® clot lysis at 30 min (Ly30) is above 10%.

This VHA treatment algorithm is based upon analysis of more than 2200 trauma subjects enrolled to a prospective observational study conducted at the participating study sites, entitled Activation of Coagulation and Inflammation in Trauma (ACIT). Analysis of the ACIT dataset has enabled the definition of clinically relevant VHA thresholds and patterns by which it is possible to rapidly identify coagulopathic patients and anticipate the need for massive transfusion. These threshold parameters have been applied to the generation of an evidence-based targeted treatment algorithm.

According to pre-designation, each study centre will only conduct VHA using either thromboelastography (TEG®) or rotational thromboelastometry (ROTEM®) to determine the following parameters:

- TEG®: RapidTEG® ACT, MA and Ly30; functional fibrinogen TEG® MA
- ROTEM®: EXTEM CT, CA5 and Li30; FIBTEM CA5

### Randomised study care – CCT arm

CCTs will be conducted for all subjects in the CCT arm at each time point up to 24 h. The tests will comprise platelet counts (PLT), activated partial thromboplastin time (aPTT), prothrombin time – international normalised ratio (PT/INR) and Clauss fibrinogen assay. PTr and Clauss fibrinogen will be measured for all study subjects at each time point.

The clinical course of subjects randomised to the CCT arm will follow a treatment algorithm utilizing CCT results (Fig. 3c) and based upon current published evidence and empiric best practice according to the PROPPR and CRASH-2 trials data (i.e. a 1:1:1 product ratio, with the anti-fibrinolytic TXA) [19–21]. The subject will be treated with standard haemostatic resuscitation based on a MTP aiming for a 1:1:1 ratio of blood components and initial TXA. In addition, the subjects will be given fibrinogen concentrate or cryoprecipitate and/or additional platelets and plasma depending on the CCT results.

Fibrinogen concentrate or cryoprecipitate will be indicated when fibrinogen values are below 2.0 g/L. Additional platelets will be indicated with PLT below 100 × 10^9^/L and additional plasma will be administrated when the INR is above 1.2 despite normal fibrinogen, defined as 2.0 g/L or above. There are no generally accepted indications for additional anti-fibrinolytic therapy using CCTs.

### Detail of outcome measures collected

#### SOFA score (Additional file 3)

SOFA score will be registered until discharge from ICU.

#### Blood products and procoagulants

Timings, total number (and doses if appropriate) of different blood products and procoagulants administered both pre-hospital and after admission to the study centre, during resuscitation and after 6 and 24 h will be recorded including:

- RBC, fresh frozen plasma (FFP)/Octaplas, cryoprecipitate, platelets, whole blood and/or autologous RBC from cell salvage
- Coagulation factor concentrates (prothrombin complex concentrate (PCC), fibrinogen, activated recombinant factor VII (rFVIIa))
- TXA

#### Fluids

Timings (during first 24 h only) and total volume of different fluids administered both pre-hospital and after admission to the study centre until 24 h will be recorded including crystalloids, colloids and hypertonic saline.

#### Thromboprophylaxis/prothrombotic medication

Type of medication administered, timings, dose and indication will be recorded daily until day 28 with particular attention to duration of treatment (stop date).

#### Bleeding episodes

Qualifying episodes will be defined by radiological evidence, like contrast extravasation on CT scan, and/or clinical suspicion, like haemodynamic instability, combined with transfusion requirement after initial haemostasis (defined as the point 1 h from the last administration of RBC and the treating clinician believes primary haemostasis has been achieved).

#### Ventilator-free days

Calculated by the subtracting the number of days spent on mechanical ventilation from 28. Death before day 28, recorded as 28.

#### Vasopressor days

Calculated as the total number of days spent on inotropic drugs, including for instance noradrenaline, dobutamine, vasopressin.

#### Renal replacement therapy days

Calculated as the number of days spent on haemodialysis or haemofiltration.

#### ICU days

The total length of stay in the ICU. If the patient is in the ICU at any time point during a calendar day, this day will be considered an ICU day.

#### Length of stay

Length of stay will be recorded in days, for the total number spent in ICU and in hospital. If the patient is in the hospital at any time point during a day, this day will be considered a hospital day.

#### Surgical episodes

Description, timing, duration and reasons for all surgical episodes will be recorded. This includes interventional radiology and bedside surgical interventions in addition to major surgical procedures.

#### Thromboembolic events

Symptomatic venous thromboembolic events will be recorded, as confirmed by either compression ultrasound/venography (DVT or by CT – pulmonary angiogram/ventilation – perfusion scan (pulmonary embolism (PE)). Other thromboembolic events, such as myocardial infarction and/or stroke, will be identified by standard clinical diagnostic investigations(s).

#### Patient disposition

First destination after discharge and disposition at 90 days post admission will be recorded as either home, rehabilitation facility, nursing home, other hospital or other.

#### Quality of life (Qol)

Subject quality of life will be assessed using the EuroQoL EQ-5D^™^ questionnaire, a standardised instrument for use as a measure of health outcome. Quality of life assessment will be conducted in the study centre upon discharge of the subject from hospital and at 90 days post admission.

The in-hospital (i.e. discharge) questionnaire will be conducted by research investigators with the patient where possible, but may also be completed with patient’s PC if necessary. The questionnaire can be completed in less than 5 min. Where the subject has already left hospital, the questionnaire will be posted out with a return stamped addressed envelope.

Patients who have not returned the questionnaire within 2 weeks of the initial request will be telephoned as a reminder to complete the questionnaire and may be asked to complete it over the telephone if necessary.

A further EuroQoL EQ-5D^TM^ questionnaire will be provided to assess subject quality of life at 90 ± 5 days post admission. Confirmation with the local (i.e. hospital care record system) and regional resources (i.e. NHS Health and Social Care Information Centre Spine Services) will ensure that only surviving patients receive a questionnaire.

### Cessation of study care (haemostasis)

For the purposes of this comparative study, haemostasis (end of study care) will be defined as the point 1 h from the last administration of RBC and the treating clinician believes that primary haemostasis has been achieved.

### Procedure for data collection

Study subject data will be captured locally using a paper Case Report Form (CRF), following local data security routines. CRF data are transferred and uploaded to a centralised study database whereupon study data integrity is reviewed weekly by the Trial Coordinating Centre.

### Adverse event reporting

Patients included in this trial have a high risk of morbidity and mortality, with either treatment being administered during a phase of critical bleeding and circulatory failure. Therefore, patients have a very high risk of experiencing several adverse events (AEs) and serious adverse events (SAEs). All SAEs, expected or not, will be recorded on a SAE form. Any SAE, death or thromboembolic or ischaemic events (myocardial infarction, stroke, pulmonary embolus, DVT) that are considered to be ‘related’ and unexpected are to be reported to the sponsor within 24 h, and to the Main Research Ethics Committee (MREC) within 15 days in line with the required timeframe.

### Urgent safety measures

The chief investigator (CI) will take urgent safety measures to ensure the safety and protection of the clinical trial subjects from any immediate hazard to their health and safety. The measures should be taken immediately. In this instance, the approval of the Ethics Committee prior to implementing these safety measures is not required. However, it is the responsibility of the CI to inform the sponsor and the Main Research Ethics Committee (MREC) (via telephone) of this event immediately.

The CI has an obligation to inform both the MREC in writing within 3 days, in the form of a substantial amendment. The sponsor (Joint Research Management Office (JRMO)) must be sent a copy of the correspondence with regards to this matter.

### Annual safety reporting

The CI will send the Annual Progress Report to the MREC using the NRES template (the anniversary date is the date on the MREC ‘favourable opinion’ letter from the MREC) and to the sponsor.

### Subject withdrawal

Every reasonable effort will be made to maintain protocol compliance and to retain patient participation in the study, consistent with the provisions of informed consent and good clinical practice. The following are potential reasons why a patient may be withdrawn from the study:

1. Withdrawal of consent/agreement
2. Retrospective exclusion: if a patient is deemed to not meet one or more of the inclusion/exclusion criteria in retrospect they will be withdrawn from the study
3. Major protocol deviation from the study design by the subject, observed or suspected by the investigator
4. Administrative: the sponsor or monitoring committees decide to terminate or discontinue the study
5. The subject’s health would be jeopardised by continued participation and hence will be withdrawn at the discretion of the investigator

The study withdrawal form will be completed for these patients and a reason for withdrawal captured. All subject’s withdrawn from the study will be managed in accordance with the hospital’s standard procedures.

### Data collection and follow-up for withdrawn subjects

Patients who withdraw from the study after randomisation will be followed for safety by conducting safety assessments through to the end of day 28. If a patient who withdraws has an ongoing SAE every effort must be made to follow up such events until satisfactory resolution is obtained or until further follow-up is no longer warranted.

### Subject replacement

Subjects who withdraw from the study will be replaced.

### End of study definition

The study will be considered closed when all surviving subjects complete in-hospital safety and outcome monitoring. This includes: safety measures of SAE rate within 28 days, total hospital stay, total critical care stays, 28-day ventilator-free days and 28-day mortality

### Statistical considerations

#### Sample size

Based upon legacy registry data from the partners, approximately 28% of patients will need massive transfusion or die. It is expected that this figure will decrease to an overall proportion of 15% in the VHA group (i.e. using VHA-guided strategy). In order to detect a difference from 28% to 15% with a power of 80% and a two-sided alpha of 0.05, 170 patients per group are required. One hundred and ninety-six patients per study arm allows a 13% dropout rate, with an allocation ratio of 1. The planned sample size for this study is 392 patients for which MTP is activated and transfusions initiated, 196 in each study arm.

#### Method of analysis

All primary and secondary outcomes will be analysed as intention-to-treat, and will include all randomised patients for whom the primary outcome of ‘alive’ and ‘free of massive transfusion’ at 24 h is recorded. The primary endpoint of patients who are alive and free of massive transfusion at 24 h will be assessed by difference in proportion with 95% confidence intervals. The chi-square test or Fischer’s exact test will be used were appropriate. Absolute risk reduction and relative risk reduction by VHA-guided therapy will be calculated.

Kaplan-Meier mortality estimates between the two arms for all-cause mortality at 6 and 24 h, as well as 28 and 90 days post admission, will be estimated for the secondary endpoint of death.

A per-protocol analysis and a sensitivity analysis will be performed for the primary endpoint. The following patients will be excluded from the per-protocol analysis:

- Patients who do not have at least one ROTEM®/TEG®/CCT test performed *and*
- Who die within 60 min after baseline blood sampling *or*
- Who achieve haemostasis within 60 min of baseline sampling

Both ROTEM®-guided and TEG®-guided therapy together (i.e. the VHA arm) will be compared with the CCT arm. Separate analyses will be performed for ROTEM®-guided and TEG®-guided therapy alone for primary endpoints and correction of coagulopathy.

All applied tests will be two-sided and *p* values of 0.05 will be accepted as statistically significant. We will report *p* values with and without correction for multiple testing.

#### Subgroup analyses

The following patient categories will be included in all primary and secondary analyses but will also be analysed separately as subgroup analyses:

- Patients with known pre-existing coagulopathy
- Oral anticoagulant therapy (except for aspirin)
- Excluding patients with severe traumatic brain injury (AIS brain 4,5 or 6)
- Patients who arriving in a coagulopathic state (PTr > 1.2)
- Patients who received a massive transfusion (10 or more RBC units in the first 24 h)

#### Sensitivity analyses

Missing data are not expected for the primary outcome. Sensitivity analysis for secondary outcomes will be assessed using 100 multiple imputations for missing data. Rubin rules will be used to summarise the results of the multiple imputations.

#### Integrated cost-effectiveness analysis

A cost-effectiveness analysis will be conducted to assess the costs and effects of VHA-guided therapy versus those of optimised empiric treatment. A model will be developed which will be structured around the key clinical time points and events in the early management pathway of bleeding trauma patients.

The two treatment policies will be compared in terms of their estimated costs and effects (quality-adjusted life years (QALYs): calculated by combining survival and HRQoL data) and incremental analyses will be performed. If VHA-guided therapy is more effective but also more costly than empirical treatment, then the incremental cost-effectiveness ratio (ICER) will be calculated. The ICER is calculated by dividing the difference in costs between VHA and empirically guided therapy by the difference in effects (QALYs) and gives the additional cost of generating one additional unit of outcome (here, a QALY).

So as to account for the uncertainty in the model input data, parameters will be entered as distributions rather than point estimates. Probabilistic sensitivity analysis will be used to take repeated random draws from all distributions simultaneously, each time recalculating the model’s results for a total of 2000 times. The uncertainty will be summarised on the cost-effectiveness plane and using cost-effectiveness acceptability curves. For each country, the modelling exercise should provide an estimate of the probability that VHA-guided therapy is likely to be cost-effective when compared with optimised empiric treatment.

### Monitoring and quality assurance

#### Summary monitoring plan

Data coordination and site management services will be performed at the sponsor institution, Queen Mary University of London. The site clinical trials coordinator will perform regular monitoring of trial documentation and CRFs.

#### Safety analysis

A pre-defined interim analysis will be performed after the enrolment of 100 patients, including an assessment of recruitment logistics with the possibility to revise the planned sample size.

A Data Safety Monitoring Board (DSMB) will review all data on outcome of the patients in the respective treatment arms. The DSMB will focus on adherence to protocol, and present pre-specified criteria that need to be fulfilled with regard to patient safety for the study to continue.

#### Audit and inspection

For the purpose of compliance with Good Clinical Practice (GCP) and Regulatory Agency Guidelines it may be necessary for the sponsor or a drug regulatory agency to conduct a site audit. This may occur at any time from the start to after conclusion of the study.

## Discussion

TIC is present early after injury in a significant proportion of patients [3–5], and is associated with increased bleeding, greater risk of complications and increased mortality, underlining the importance of early detection and aggressive treatment [4, 6, 7, 22].

Improvements in transfusion strategies over the last decade are associated with better outcome [23–26]. The results of the PROPPR trial [19] provide the best evidence to date for ratio-based trauma resuscitation. In that study, a MTP aiming at a 1:1:1 ratio of plasma 1: platelets 1: RBC 1 until haemorrhage control was associated with better outcome than a 1:1:2 ratio. However, PROPPR did not allow adjustments in the individual treatment based on results from coagulation tests during the course of resuscitation.

Traditionally, CCTs such as prothrombin time/international normalised ratio (PT/INR), activated partial thromboplastin time (aPTT), fibrinogen concentration and PLT have been recommended to guide resuscitation in bleeding trauma patients [27]. However, none of the existing CCTs have proven to be robust in detecting TIC or predicting massive transfusion. Moreover, CCTs are time-consuming laboratory tests only reflecting the initial steps of blood coagulation and not taking into account the interaction between platelets and coagulation factors.

On that background, the potential benefit of Viscoelastic Haemostatic Assays (VHAs), such as TEG® and ROTEM®, in the trauma setting has gained much attention over the last decade. VHAs are dynamic tests; they may be performed bedside with their first results available within minutes of initiation. Several algorithms for guiding resuscitation in bleeding trauma patients based on VHA parameters have been published [28, 29]. None of them have been developed based on real-time large cohorts of trauma patients.

The updated European guidelines addressing the management of bleeding and coagulopathy following major trauma recommend the use of viscoelastic methods to assist in characterising the coagulopathy and in guiding haemostatic therapy [27] although the evidence to support the use of VHAs in this category of patients is insufficient [15–18].

Based on limited existing evidence to support the use of VHA versus CCTs in monitoring the resuscitation of massively bleeding trauma patients, our aim is to evaluate the differences between VHA-guided and CCT-guided transfusion in trauma patients, and to create robust evidence-based guidelines for massive transfusion in trauma patients.

iTACTIC has obvious limitations, based on the actual level of evidence in this field. The challenges include the heterogeneity of, and access to, a population of severely injured patients as well as the development of relevant algorithms. Strength and weaknesses will be fully addressed when the trial results are published.

### Trial status

This study is ongoing and started recruiting June 2016. Recruitment will be completed mid 2018.

## Additional files

Additional file 1: Protocol version 3.0/14.03.2017. (PDF 2172 kb)

Additional file 2: SPIRIT Checklist. (DOC 129 kb)

Additional file 3: SOFA score. (ZIP 217 kb)

## Abbreviations

ACIT: Activation of Coagulation and Inflammation in Trauma
ACT (ROTEM®): Activated clotting time (ROTEM®)
AE: Adverse event
APTT: Activated partial thromboplastin time
ATLS®: Advanced Trauma Life Support
CA 5 (ROTEM®): Clot Amplitude at 5 min (ROTEM®)
CCT: Conventional coagulation tests
CI: Chief investigator
CONSORT: Consolidated Standards of Reporting Trials
CRF: Case Report Form
CT: Computer tomography
CT (ROTEM®): Clotting time (ROTEM®)
DSMB: Data Safety Monitoring Board
DVT: Deep venous thrombosis
ED: Emergency department
FAST: Focussed Assessment with Sonography in Trauma
FF (TEG®): Functional fibrinogen (TEG®)
FFP: Fresh frozen plasma
GCP: Good Clinical Practice
ICER: Incremental cost-effectiveness ratio
ICU: Intensive care unit
JRMO: Joint Research Management Office
LI30 (ROTEM®): Lysis Index at 30 min (ROTEM®)
(TEG®): Clot lysis at 30 min (TEG®)
MA: Maximum Amplitude
MREC: Main Research Ethics Committee
MTP: Massive transfusion protocol
PC: Personal consultee
PCC: Prothrombin complex concentrate
PE: Pulmonary embolism
PROPPR: Pragmatic, Randomised Optimal Platelet and Plasma Ratios trial
PTr: Prothrombin Time/International Ratio (PT/INR)
QALY: Quality-adjusted Life Years
QMUL: Queen Mary University of London
QoL: Quality of life
RBC: Red blood cells
RCT: Randomised controlled trial
rFVIIa: Activated recombinant factor VII
ROTEM®: Rotational thromboelastometry
SAE: Serious adverse event
SOFA: Sequential Organ Failure Assessment
SPIRIT: Standard Protocol Items: Recommendations for Interventional Trials
Subject: An individual who takes part in a clinical trial
TEG®: Thromboelastography
TIC: Trauma-induced coagulopathy
TXA: Tranexamic acid
VHA: Viscoelastic Haemostatic Assays
