# Supplementary material for: iTACTIC – implementing Treatment Algorithms for the Correction of Trauma-Induced Coagulopathy: study protocol for a multicentre, randomised controlled trial
Source: Trials. 2017 Oct 18;18:486. doi: 10.1186/s13063-017-2224-9 (PMC5648415; doi:10.1186/s13063-017-2224-9)
Supplement: Supplementary file 1 — Protocol version 3.0/14.03.2017. (PDF 2172 kb) [file 13063_2017_2224_MOESM1_ESM.pdf]

# iTACTIC: Implementing Treatment Algorithms for the Correction of Trauma Induced Coagulopathy

A multi-centre, prospective, randomized controlled study to compare outcomes of viscoelastic haemostatic assay (VHA)-guided resuscitation versus optimised conventional coagulation test (CCT) resuscitation support in haemorrhaging trauma patients.

# CLINICAL TRIAL PROTOCOL

## BARTS HEALTH NHS TRUST

| | NUMBER | DATE |
|---|---|---|
| VERSION | 3.0 | 14/03/2017 |
| AMENDMENTS | | |

# Implementing Treatment Algorithms for the Correction of Trauma Induced Coagulopathy

A multi-centre, prospective, randomized controlled study to compare outcomes of viscoelastic haemostatic assay (VHA)-guided resuscitation versus optimised conventional coagulation test resuscitation support in haemorrhaging trauma patients.

| | |
|---|---|
| **Short title/Acronym:** | iTACTIC |
| **Sponsor** | Queen Mary, University of London<br><br>*Contact person of the above sponsor organisations is:*<br><br>Dr Sally Burtles<br>Director of Research Services & Business Development<br>Joint Research Management Office<br>QM Innovation Building<br>5 Walden Street<br>London E1 2EF<br>Phone: 020 7882 7260<br>Email: sponsorsrep@bartshealth.nhs.uk |
| **REC Reference** | 16/LO/0004 |
| **Chief Investigator:** | Professor Karim Brohi<br>Centre for Trauma Sciences, Blizard Institute, Queen Mary University of London, 4 Newark Street, Whitechapel, London, E1 12AT, UK<br>Phone: +44 77 0319 0545<br>Email: k.brohi@qmul.ac.uk |
| **REC reference:** | 16/LO/0004 |
| **EudraCT reference:** | *Insert once known* |
| **Study sites:** | Academic Medical Centre, Amsterdam,<br>The Netherlands |

Kliniken der Stadt Köln gGmbH, Cologne, Germany

Rigshospitalet (Copenhagen University Hospital), Copenhagen, Denmark

John Radcliff Hospital, Oxford, United Kingdom

Oslo University Hospital, Oslo, Norway

The Royal London Hospital, London, United Kingdom

Centre for Trauma Sciences, Blizard Institute, Queen Mary University of London, London, United Kingdom

Health Economics Research Centre, Nuffield Department of Population Health, University of Oxford, Oxford, United Kingdom

Nottingham University Hospitals Queens Medical Centre, Nottingham, UK

Radboud university medical center, Nijmegen, Netherlands

University of Kansas Hospital (UKH), Kansas City, USA

## CONTENTS PAGE


1. **GLOSSARY OF TERMS AND ABBREVIATIONS** . . . 6
2. **SIGNATURE PAGE** . . . . . . . . 8
3. **SUMMARY** . . . . . . . . . 9
4. **INTRODUCTION** . . . . . . . . 13
   4.1. Background . . . . . . . . 13
   4.2. Clinical data . . . . . . . . 13
   4.3. Rationale and Risk/Benefits . . . . . . 14
5. **TRIAL OBJECTIVES** . . . . . . . 15
   5.1. Primary objective . . . . . . . 15
   5.2. Secondary objective . . . . . . 15
   5.3. Primary endpoint . . . . . . . 15
   5.4. Secondary endpoints . . . . . . . 15
6. **METHODOLOGY** . . . . . . . . 17
   6.1. Inclusion criteria . . . . . . . 17
   6.2. Exclusion criteria . . . . . . . 17
   6.3. Study design . . . . . . . . 17
   6.4. Study scheme diagram . . . . . . . 18
7. **STUDY PROCEDURES** . . . . . . . 19
   7.1. Screening procedures . . . . . . . 19
   7.2. Randomisation procedures . . . . . . 19
   7.3. Schedule of intervention . . . . . . . 19
   7.4. Procedures . . . . . . . . . 22
   7.5. Study intervention . . . . . . . . 26
   7.6. Procedure for data collection . . . . . . 31
   7.7. Adverse events reporting . . . . . . . 32
   7.8. Investigators assessment . . . . . . . 35
   7.9. Safety analysis . . . . . . . . 36
   7.10. Subject withdrawal . . . . . . . 36
   7.11. Data collection and follow-up for withdrawn subjects . 36
   7.12. Subject replacement . . . . . . 36
   7.13. Schedule of intervention (in diagrammatic format) . 37
   7.14. End of study definition . . . . . . 38

**8. STATISTICAL CONSIDERATIONS** . . . . . 38
8.1. Sample size . . . . . . . 38
8.2. Method of analysis . . . . . . . 38
**9. ETHICS** . . . . . . . . 40
9.1. Safety considerations . . . . . . 40
9.2. Risks . . . . . . . . 40
9.3. Benefit to the patient . . . . . . 41
9.4. Benefit to society . . . . . . . 41
9.5. Patient enrolment . . . . . . . 42
**10. DATA HANDLING AND RECORD KEEPING** . . . 44
10.1. Confidentiality . . . . . . 44
10.2. Study documents . . . . . . 45
10.3. Case report form . . . . . . 46
10.4. Identification of source data . . . . 46
10.5. Record retention and archiving . . . . 47
10.6. Compliance . . . . . . . 47
10.7. Clinical governance issues . . . . . 47
**11. QUALITY CONTROL AND QUALITY ASSURANCE** . . 48
11.1. Summary monitoring plan . . . . . 48
11.2. Audit and inspection . . . . . 49
11.3. Serious breaches in GCP or trial protocol . . 49
11.4. Non-compliance . . . . . . 49
11.5. Sponsors termination of study . . . . 50
11.6. Indemnity and insurance . . . . . 50
11.7. Post-trial care . . . . . . 50
**12. APPENDICES** . . . . . . . 51
12.1. References . . . . . . . 51
12.2. SOFA table of organ dysfunction . . . . 52
12.3. List of management documents . . . . 52
12.4. Core lab instructions to investigators . . . 53
12.5. Definitions of transfusion reactions . . . 54

# 1. GLOSSARY OF TERMS AND ABBREVIATIONS

| | |
|---|---|
| **APTT** | Activated Partial Thromboplastin Time |
| **AE** | Adverse Event |
| **AR** | Adverse Reaction |
| **AUC** | Area Under the Curve |
| **CCT** | Conventional Coagulation Tests |
| **CI** | Chief Investigator |
| **CRF** | Case Report Form |
| **CTIMP** | Clinical Trial of an Investigational Medicinal Product |
| **DMC** | Data Monitoring Committee |
| **EC** | European Commission |
| **EMEA** | European Medicines Agency |
| **EU** | European Union |
| **EudraCT** | European Union Drug Regulating Authorities Clinical Trials |
| **FiO2** | Fraction of inspired Oxygen |
| **FFP** | Fresh Frozen Plasma |
| **PaO2** | Partial pressure of arterial Oxygenation |
| **GCP** | Good Clinical Practice |
| **GCS** | Glasgow Coma Score |
| **ICU** | Intensive Care Unit |
| **ISRCTN** | International Standard Randomised Controlled Trial Number |
| **JRMO** | Joint Research Management Office |
| **MTP** | Massive Transfusion Protocol |
| **PC** | Personal Consultee |
| **PI** | Principle Investigator |
| **PTr** | Prothrombin Time / International Ratio (PT/INR) |
| **QALY** | Quality Adjusted Life Years |
| **QMUL** | Queen Mary University of London |
| **QoL** | Quality of Life |
| **RBC** | Red Blood Cells |
| **RCT** | Randomised Controlled Trial |
| **REC** | Research Ethics Committee |
| **ROTEM** | Rotational Thromboelastometry |
| **RRR** | Relative Risk Reduction |

| | |
|---|---|
| **SAE** | Serious Adverse Event |
| **SAR** | Serious Adverse Reaction |
| **SOFA** | Sequential Organ Failure Assessment |
| **SOP** | Standard Operating Procedure |
| **Subject** | An individual who takes part in a clinical trial |
| **SUSAR** | Suspected Unexpected Serious Adverse Reaction |
| **TEG** | Thromboelastography |
| **TIC** | Trauma Induced Coagulopathy |
| **VHA** | Viscoelastic Haemostatic Assays |

## 2. SIGNATURE PAGE

**Chief Investigator Agreement**

The clinical study as detailed within this research protocol **(Version 3.0, dated 14 Mar 17)**, or any subsequent amendments will be conducted in accordance with the Research Governance Framework for Health & Social Care (2005), the World Medical Association Declaration of Helsinki (1996) and the current applicable regulatory requirements and any subsequent amendments of the appropriate regulations.

**Chief Investigator Name:** Professor Karim Brohi

**Chief Investigator Site:** Royal London Hospital, Barts Health NHS Trust

**Signature and Date:**

**Principal Investigator Agreement** *(if different from Chief investigator)*

The clinical study as detailed within this research protocol **(Version 3.0, dated 14 Mar 17)**, or any subsequent amendments will be conducted in accordance with the Research Governance Framework for Health & Social Care (2005), the World Medical Association Declaration of Helsinki (1996) and the current applicable regulatory requirements and any subsequent amendments of the appropriate regulations.

**Principal Investigator Name:** Professor Karim Brohi

**Principal Investigator Site:** Royal London Hospital, Barts Health NHS Trust

**Signature and Date:**

# 3. SUMMARY

## General Information

This document was constructed using the Non-CTIMP Protocol Template (V3.0, 15 Nov 2012 Final JMRO Master Template). It describes a comparison of viscoelastic haemostatic assay-led resuscitation versus conventional resuscitation support in haemorrhaging trauma patients in a Phase 2a multi-site randomized control trial, and provides information for entering patients/subjects into it. The protocol should not be used as a guide for the treatment of other patients. Every care has been taken in drafting this protocol, but corrections or amendments may be necessary. These will be circulated to the registered investigators in the trial, but those entering participants for the first time are advised to contact the Trial Manager to confirm they have the most up to date version.

## Compliance

The trial will be conducted in compliance with the approved protocol, the Declaration of Helsinki (2008) the Principles of Good Clinical Practice (GCP), European Commission Directive 2005/28/EC with the implementation in national legislation in the UK by Statutory Instrument 2004/1031 and subsequent amendments, the UK Data Protection Act and the National Health Service Research Governance Framework for Health and Social Care.

## Sponsor

Queen Mary University of London (QMUL) is the trial sponsor. Queries relating to the QMUL sponsorship of the trial should be addressed to the Director of Research Services & Business Development, Dr Sally Burtles, JRMO, QMUL Innovation Department, 5 Walden Street, London, E1 2EF, E-mail: sponsorsresp@bartshealth.nhs.uk, or via the trial manager.

## Funding

This trial is part-funded by the European Commission under the HEALTH-Contract No. F3-2013-602771, entitled “Targeted Action for Curing Trauma Induced Coagulopathy” (TACTIC).

| | |
|---|---|
| TITLE | A multi-centre, prospective, randomized controlled study to compare outcomes of viscoelastic haemostatic assay (VHA)-guided resuscitation versus optimised conventional coagulation test resuscitation support in haemorrhaging trauma patients. |
| SHORT TITLE | Implementing Treatment Algorithms for the Correction of Trauma Induced Coagulopathy (iTACTIC) |
| PROTOCOL VERSION/DATE | v3.0 / 14/03/2017 |
| METHODOLOGY | Non-blinded, randomised controlled trial |
| STUDY DURATION | The study will enrol patients over a 24-month period. Patient follow-up, data cleaning & analysis will take a further 9 months. |
| TREATMENT DURATION | The subject's participation in the study will last a maximum of 28 days. |
| STUDY LEADS | CHIEF INVESTIGATOR:<br>Prof Karim Brohi<br>Centre for Trauma Sciences, Blizard Institute<br>Queen Mary University of London,<br>4 Newark Street, London, E1 2AT<br>Phone: +44 7703 190545<br>Email: k.brohi@qmul.ac.uk<br><br>CO-INVESTIGATOR:<br>Dr Christine Gaarder<br>Department of Traumatology, Oslo University Hospital,<br>166 Kirkeveien, Oslo, NO-0424<br>Phone: +47 4131 8992<br>Email: tinagaa@ous-hf.no |
| PRIMARY OBJECTIVE | The primary objective is to compare the haemostatic effect of Viscoelastic Haemostatic Assays (VHA)-guided transfusion strategy versus optimized non-VHA guided transfusion strategy in haemorrhaging trauma patients. |
| SECONDARY OBJECTIVES | The secondary objectives of the study are to determine the effects of VHA-led versus optimized non-VHA guided resuscitation on organ failure, hospital stay, critical care stay, health care resource needs and mortality. |
| PHASE OF THE TRIAL | Phase 2a |

| | |
|---|---|
| **NUMBER OF SUBJECTS** | A total target of 392 adult male and female severely injured trauma patients with ongoing traumatic haemorrhage. |
| **INCLUSION CRITERIA** | **INCLUSION CRITERIA**<br>A patient will be eligible for the study if they meet the following criteria:<br>• Adult trauma patients (according to local definitions)<br>• Present with clinical signs of haemorrhagic shock<br>AND<br>• Activate the local massive haemorrhage protocol and initiate first transfusion<br>• Randomised within 3 hours of injury and 1 hour of admission to the emergency department of the participating study site<br>• Agreement is provided on behalf of incapacitated patients by Personal Consultee or Nominated Consultee (i.e. trauma team leader) |
| **STATISTICAL METHODOLOGY AND ANALYSIS** | **ANALYSIS POPULATIONS:**<br>The analysis populations will be defined as follows:<br>*Intention to treat analysis*<br>All patients randomized will be analysed according to the treatment arm to which they are assigned.<br>*Per protocol analysis*<br>The following patients will be excluded from the per protocol analysis:<br>• Patients who do not have at least one VHA or Conventional Coagulation Test (CCT) performed<br>• Patients who die within 60 minutes after baseline blood sampling<br>• Patients who achieve haemostasis within 60 minutes of baseline sampling.<br>**PRIMARY ENDPOINT ANALYSIS:**<br>Difference in proportion will be examined with the Chi-square test. Absolute and Relative Risk Reductions will be calculated.<br>**SECONDARY VARIABLES ANALYSES:**<br>Difference in proportions will be examined with the Chi-square test. Differences between continuous variables will be assessed by difference in means and the Students-t test, or |

| | |
|---|---|
| | difference in median and the Mann-Whitney U test as appropriate. |
| **Proposed Start Date** | 1st December 2015 |
| **Proposed End Date** | 30th November 2017 |
| **Study Duration** | 24 months |

**SAE REPORTING**

Within 24 hours of becoming aware of a reportable SAE please fax a completed SAE form to the Sponsor on:

+44 20 7882 7276

Or E-mail information to: **research.safety@bartshealth.nhs.uk**

# 4. INTRODUCTION

## 4.1 BACKGROUND

Traumatic injury is responsible for a large and increasing proportion of the world's burden of disease and is the 4th leading cause of death globally [1]. Half of all trauma deaths are due to bleeding and most of these will occur within 6 hours from injury [2]. Hemorrhagic shock following injury has been shown to induce a clotting dysfunction (i.e. coagulopathy) within minutes [3-5].

Such early trauma induced coagulopathy (TIC) may exacerbate bleeding and is associated with higher mortality and morbidity [4,6,7]. Many more injured patients will go on to develop different types of coagulopathy at different times during the course of their treatment, either as a result of their body's on-going response to trauma or as a consequence of their clinical care. Ultimately coagulopathic, hemorrhaging trauma patients have increased blood transfusion requirements, increased mortality and more adverse outcomes [8].

Despite improvements in surgical techniques, resuscitation strategies and intensive care treatments, outcomes for critically injured patients remain poor with severe bleeding, brain injury, tissue damage and multiple organ failure linked to high mortality [9]. Within the last decade research focusing on TIC has led to improved resuscitation strategies, resulting in the early and more aggressive use of blood products and coagulation factors for the management of massively bleeding patients.

## 4.2 CLINICAL DATA

In spite of improved resuscitation strategies, current transfusion therapy still fails to correct coagulopathy during ongoing haemorrhage [10]. The mechanisms and genesis of TIC have yet to be fully elucidated, and there are many questions around how to optimally diagnose, resuscitate and monitor the critically bleeding trauma patient.

It is important to detect TIC as early as possible. Conventional coagulation tests (CCT), such as prothrombin time/international ratio (PT/INR), activated partial thromboplastin time (APTT), fibrinogen concentration and platelet counts, have traditionally been used. However, there is a striking lack of evidence to support the use of these CCT to monitor resuscitation, although threshold triggers for intervention based on CCT have been suggested [5].

Recent published evidence describes an increasing recognition for the potential of the two current market-leading Viscoelastic Haemostatic Assays (VHAs) namely Thromboelastography (TEG®; Haemonetics Incorporation) and Rotational Thromboelastometry (ROTEM®; TEM Innovation GmbH). Both platforms use similar test modes to rapidly and accurately determine the functional coagulation status of patient whole blood *(see Figure 1)*.

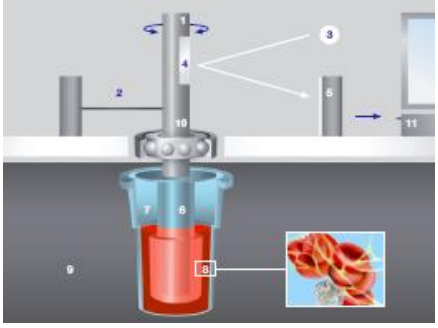


***Figure 1* – Viscoelastic Haemostatic Assay (VHA) systems***: In TEG a pin attached to a torsion wire is immersed in an oscillating cup containing the blood sample at 37ºC. Conversely in ROTEM the cup is stationary and the oscillating pin is attached to an optical detector. As clot forms, the gap between cup and pin is bridged and the oscillation is transmitted from the cup to the pin (TEG), or impedes rotation of the pin (ROTEM).*

The relative contribution of blood components such as fibrinogen and platelets to clot strength can be evaluated through the use of specific inhibitors or agonists. The viscoelastic properties of blood samples are recorded under low shear conditions, thereby providing a comprehensive visual profile (see *Figure 2*) of clot formation and breakdown (fibrinolysis).

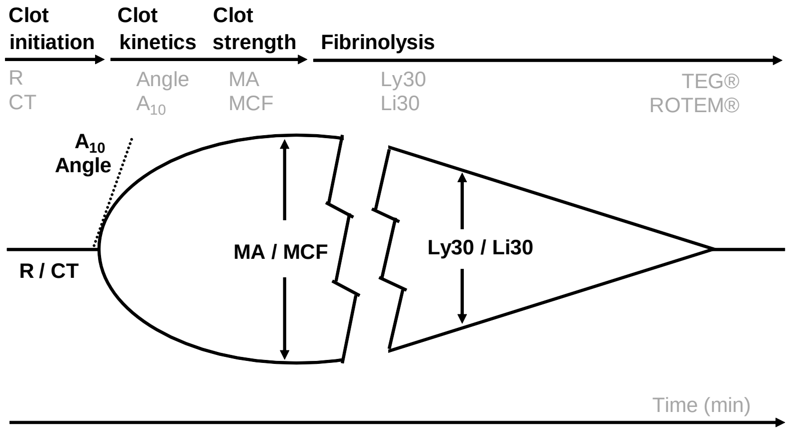


***<u>Figure 2</u> – TEG® and ROTEM® measurements.*** *Oscillation is detected and a characteristic trace generated, with a profile reflecting different aspects (parameters) of coagulation: TEG®: R, Reaction time; Angle; MA, Maximum Amplitude; Ly30, hyperfibrinolysis after 30 min. ROTEM®: CT, Clotting Time; A10;, Amplitude after 10 min; MCF, Maximum Clot Firmness; Li30, hyperfibrinolysis after 30 min.*

VHA may be performed at the point-of-care to provide clinically relevant results within a 5-10 minute timeframe and thus may be repeated in a massive bleeding situation to guide the transfusion. Furthermore as well as the benefit of rapid readout, VHAs provide a functional description of coagulation status unlike existing CCTs (e.g. the potential to detect hyperfibrinolysis).

Whilst VHA has been used for many years in liver transplant and cardio-pulmonary surgery, there remains the absence of robust data supporting its universal uptake in the context of trauma. Whilst some publications have attempted to identify VHA-patterns and thresholds characterizing TIC and need for massive transfusion in trauma patients, definitive evidence proving its superiority over CCTs in the diagnosis and management of coagulopathy in the acute setting is not conclusive [11,12].

## 4.3 RATIONALE AND RISKS/BENEFITS

Although considered a preventable major cause of death, the management of coagulopathic bleeding in trauma patients remains primarily based upon retrospective registry studies of survival and extrapolating the results of transfusion practice performed in the elective, non-acute surgical setting. Treatment is diverse comprising the empiric transfusion of red blood cells (RBC) and clotting product supplements to patients, blind to the type and severity of TIC they may have - or indeed even if they do not have coagulopathy.

It is well established that blood transfusion carries significant health risks both related to transmission of pathogens and to the development of transfusion reactions. Published in 2015, the results of the Pragmatic, Randomized Optimal Platelet and Plasma Ratios (PROPPR) trial [13] provide the best evidence to date for optimal

trauma haemorrhage resuscitation. PROPPR demonstrated that an empiric massive transfusion protocol (MTP) aiming at ratio 1:1:1 of blood components (RBC 1: plasma 1: platelets 1) administered from the early phase of care and during on-going haemorrhage was associated with better outcome than a 1:1:2 ratio.

This prospective randomized controlled trial (RCT) will employ evidence-based treatment algorithms to compare outcomes of VHA-guided resuscitation versus CCT resuscitation support in haemorrhaging trauma patients.

The hypothesis for this comparative study is that VHA-directed therapy will enhance early hemostatic control by the targeted correction of TIC, whilst also reducing the blind administration of blood products and procoagulants to all bleeding trauma patients, including those not having TIC. This would significantly reduce both the number of patients receiving blood transfusion and the number of transfused blood products per transfused patient, thereby improving both patient safety and resource utilization.

# 5. TRIAL OBJECTIVES

## 5.1 PRIMARY OBJECTIVE

The primary objective is to compare the haemostatic effect of viscoelastic haemostatic assay-guided transfusion strategy versus optimized CCT guided transfusion strategy in haemorrhaging trauma patients.

## 5.2 SECONDARY OBJECTIVE

The secondary objectives of the study are to determine the effects of VHA-led versus optimized CCT guided resuscitation on organ failure, hospital stay, critical care stay, health care resource needs and mortality.

## 5.3 PRIMARY ENDPOINT

The primary endpoint is the proportion of subjects alive and free of massive transfusion* at 24 hours.

** receiving 10 or more units of RBC within 24 hours*

## 5.4 SECONDARY ENDPOINTS

The secondary end points listed below will be analysed in order to provide a sensitive and comprehensive description of outcomes and healthcare resource demands for the VHA and CCT arm subjects:

- All-cause mortality at 6 and 24-hours and 28 & 90-days post admission
- Duration and severity of coagulopathy until haemostasis, as defined by the area under the time[1] multiplied by PTr curve [2,3]
- Proportion of patients who have corrected coagulopathy after first 8 units of RBC
- Time to haemostasis[1]

- Time spent in coagulopathic condition until haemostasis[1]
- Blood products (RBC, plasma, platelets alone and in total) first 6 and 24 hours after admission
- 28-day ventilator free days
- 28-day ICU-free days
- Total hospital length of stay
- 28-day symptomatic thromboembolic events
- Incidence of transfusion related complications
- Incidence of organ dysfunction
- Health care resource, productivity costs and HRQoL (EuroQol EQ-5D™ at discharge or day 28, and at day 90)
- Lifetime health economic cost-effectiveness of personalized VHA-guided haemorrhagic treatment versus MTP-based on best practice and CCT

[1]*Time of haemostasis is defined as having occurred at the end of the first hour free of red cell transfusions and the treating clinicians believe primary haemostasis has been achieved*

[2]*Coagulopathy defined as PTr >1.2*

[3]*Patients who die will have their time of haemostasis set at 24 hours, and last PTr extrapolated to this time point.*

Note: All non-survivors (patients who die during the 28-day study period) will receive 0 days for Hospital-free days.

# 6. METHODOLOGY

## 6.1 INCLUSION CRITERIA

Adult trauma patients (according to local definitions) will be enrolled if they satisfy each of these inclusion criteria:

- Present with clinical signs of haemorrhagic shock
- Activate the local massive haemorrhage protocol and initiate first transfusion
- Randomised within 3 hours of injury and 1 hour of admission to the ED of the participating study site
- Agreement is provided on behalf of incapacitated patients by Personal Consultee or Nominated Consultee (i.e. trauma team leader)

## 6.2 EXCLUSION CRITERIA

Patients will be ineligible to be enrolled in the study if:

- Any inclusion criteria are not met

## 6.3 STUDY DESIGN

This randomised controlled study will follow the clinical course of haemorrhaging trauma patients on admission to the ED and for up to 28 days thereafter. Only injured patients suffering haemorrhage and shock will be considered for enrolment into the study which will, of necessity, include patients who are unable to give consent for themselves (see *Section 9.5.1*).

CCT
The CCT arm in this randomised controlled trial will comprise treatment according to an optimized MTP guided by CCTs (see *Section 7.5.4*) based upon current published evidence and empiric best practice according to the PROPPR and CRASH-2 trials data (i.e. 1:1:1 product ratio, with the anti-fibrinolytic Tranexamic Acid (TXA)) [13-15].

VHA
The VHA arm will employ an evidence-based algorithm for VHA-directed treatment (see *Section 7.5.5*) based upon current published evidence and empiric best practice according to the PROPPR and CRASH-2 trials data (i.e. 1:1:1 product ratio, with the anti-fibrinolytic Tranexamic Acid (TXA)) [13-15].

This VHA treatment algorithm is based upon analysis of more than 2,200 trauma subjects enrolled to a prospective observational study conducted at the participating study sites, entitled Activation of Coagulation and Inflammation in Trauma (ACIT) [10]. Analysis of the ACIT dataset has enabled the definition of clinically-relevant VHA thresholds and patterns by which it is possible to rapidly identify coagulopathic patients and anticipate the need for massive transfusion. These threshold parameters have been applied to the generation of an evidence–based targeted treatment algorithm, which will be used to treat the interventional group and compared with patients receiving optimized MTP care alone.

## 6.4 STUDY SCHEME

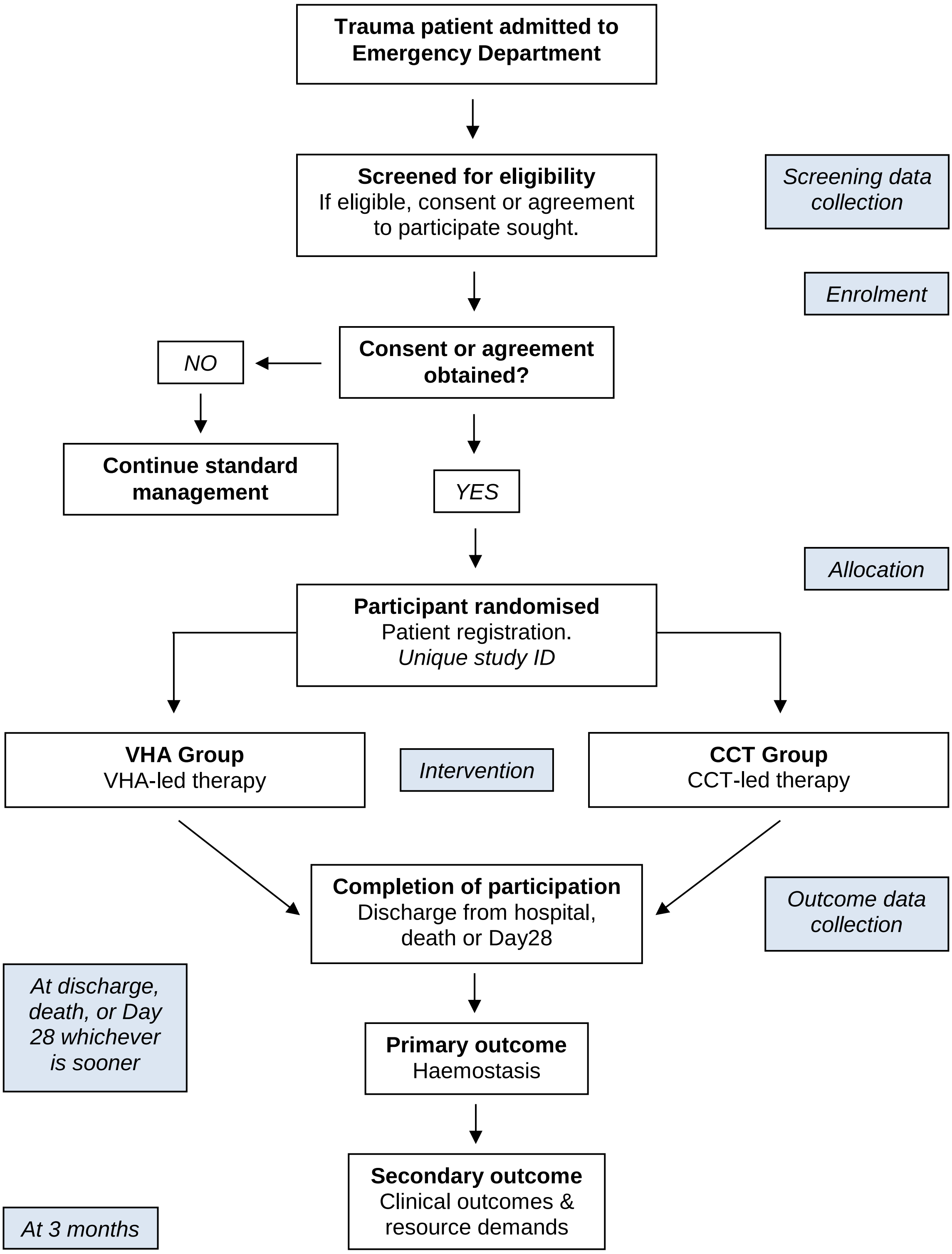

# 7. STUDY PROCEDURES

## 7.1 SCREENING PROCEDURES

It will be the responsibility of the local investigator(s) to identify eligible adult trauma patients with haemorrhagic shock and ongoing bleeding as soon as possible after the patient has arrived in the ED, using local transfusion triggers.

If patients are deemed to be eligible, consent for entry into the study will be sought (see *Section 9.5*).

Data will be collected on all adult trauma patients admitted to the ED with major blood loss throughout the recruitment period, who are screened for eligibility by the investigator(s).

A screening log will be completed once a week, which will record all patients considered for eligibility to the trial. The log will include age, gender, inclusion/exclusion criteria and other reasons for non-enrolment. The screening log will include patients approached but for whom consent was not obtained for the trial (with reasons). The screening log data will be reviewed at regular intervals.

## 7.2 RANDOMISATION PROCEDURES

Enrolled patients will be block randomized per centre to either the CCT or the VHA study arm. The randomised controlled trial will be unblinded.

Once a patient is determined eligible for the study and informed consent or agreement has been obtained, each subject will be enrolled as soon as possible and will be assigned a unique study identifier; this will be used throughout the subjects' participation in the study and will be documented on the enrolment log. This unique identifier will be alphanumeric, reflecting the study, the site and commencing at '001', ascending sequentially thereafter (i.e. iTACTIC_RLH_001 etc)

Randomisation will occur within 3 hours of injury and within one hour of admission.

24-hour on-site randomization will be performed by envelope opening, to allow for immediate allocation of subjects. An independent party, appointed by the Sponsor, will generate the randomization sequence and site envelopes centrally. These will be provided to each study site in a secure manner.

The site Investigator or designee will open a pre-sealed envelope containing the randomised treatment group allocation. Once randomised, the subject's pack details will be documented on the enrolment log.

## 7.3 SCHEDULE OF INTERVENTION

The following procedures will be conducted after arrival of the subject at the hospital:

### 7.3.1 Screening

- Assess eligibility (refer to inclusion/exclusion criteria)
- Check that written consent/agreement has been given to participate in the study in the form of a signed and witnessed informed consent form

- Medical and trauma history
- Physical examination
- Medical and treatment-related events prior to randomization, as well as surgical interventions
- All fluid/ transfusions and tranexamic acid given prior to randomization
- Resuscitate in accordance with the randomization sequence

### 7.3.2 Resuscitation

The following assessments will be conducted at baseline, after every 4th unit RBC during on-going resuscitation and until haemostasis:

- Physical examination (at baseline and haemostasis timepoints only)
- Blood sampling
- Haematology (baseline only)
- Blood chemistry (baseline only)
- Arterial blood tests (at baseline and haemostasis timepoints only)
- CCT
- VHA parameters in VHA arm

### 7.3.3 Hours 6 and 24 hours

The following assessments will be conducted at each specified time point after admission:

- Haematology (24 hours only)
- Arterial blood tests
- Blood chemistry (24 hours only)
- CCT
- VHA parameters in VHA arm
- Total blood products
- Total coagulation factor concentrates and Tranexamic acid
- Timings and total fluid volume
- Serious adverse events (SAE)
- Medical and treatment events, as well as surgical interventions
- Time of haemostasis
- Time of corrected coagulopathy

### 7.3.4 Day 0 to Day 28

The following assessments will be conducted daily, until 28 days post admission (inclusive) or upon discharge or death:

- Surgical treatment
- Thromboprophylaxis/prothrombotic medication
- Inotropes
- Episodes of bleeding
- Symptomatic thrombotic events
- Organ failure assessment (SOFA score) until discharge from ICU
- Serious adverse events (SAE)
- Death

#### 7.3.5 Day 28 or upon hospital discharge

The following assessments will be recorded on day 28 or upon discharge from hospital, whichever occurs sooner.

- Days on mechanical ventilation
- Days on vasopressors
- Days on renal replacement therapy
- Total length of stay in the intensive care unit
- Total length of stay in the hospital
- Death
- Other SAEs
- Symptomatic venous thromboembolic events

Note - If the patient is discharged before Day 28, the patient will be examined for SAEs up to Day 28 at their Day 90 ± 5 days assessment.

#### 7.3.6 Upon hospital discharge

The following assessments will be upon discharge from hospital, or within 5 days thereafter:

- QoL questionnaire (performed on hospital discharge or day 28, whichever comes first)
- First destination after discharge (home, rehabilitation facility, nursing home, other hospital, other)

#### 7.3.7 Day 90

The following assessments will be conducted on day 90 ± 5 days:

- Mortality status
- QoL questionnaire

- Current disposition (home, rehabilitation facility/nursing home, other hospital, other)

## 7.4 PROCEDURES

### 7.4.1 Medical and trauma history

The medical and trauma history will be conducted at screening and should include (but not be limited to) date & estimated time of injury, injury description, abbreviated injury scale (AIS by body region), date and time of admission to hospital, subjects' medical history with an emphasis on the trauma, current medication, past medication and allergies. Known pregnancy, any pre-injury coagulopathy, use of anticoagulant medication (excluding aspirin) and reason for use will be specifically documented in the CRF. This will be ascertained as part of the subjects' initial clinical assessment by the trauma team and the investigator.

The known medical history must be documented in the subject's notes (the on-site source document) prior to randomised care and also recorded in the CRF. Any further medical history ascertained subsequent to the initial assessment will be derived from the subjects' notes during the assessment phase.

### 7.4.2 Physical examination

On-scene observations and observations made upon arrival at the ED and should include (but not limited to) age, weight, height, Glasgow Coma Scale (GCS), Heart Rate (HR), Systolic Blood Pressure (SBP), Glasgow Coma Score (GCS), Respiratory Rate (RR), Arterial Oxygen fraction-ratio ($PaO_2/FiO_2$ ratio), Oxygen Saturation ($SaO_2$), body temperature.

SBP will be recorded using a standard sphygmomanometer reading or by invasive arterial monitoring (where this has been inserted as part of routine clinical care). Pulse rate and $SaO_2$ will be measured by pulse oximetry or by arterial monitoring as above. Respiration rate will be assessed by observation as breaths per minute and a tympanic body temperature measure will be used, unless other methods (e.g. rectal or urinary bladder monitoring) are in place for continuous clinical monitoring. GCS will be recorded from the subject notes or assessed clinically.

### 7.4.3 Blood samples

During the first 24 hours, subjects will have a maximum of 20 mls of blood drawn per sampling time point (see *Section 12.4*) to determine the level of key blood components and functional coagulation status at the following time points:

- At hour 0 (within 10 minutes) immediately following study enrolment
- After every 4$^{th}$ unit RBC transfused until haemostasis (maximum 10 mls blood obtained at RBC time points)
- At haemostasis (within 30 minutes)
- At 6 hours post admission (within 30 minutes)
- At 24 $\pm$ 1 hours post admission

The maximum volume of 20 mls of blood sampled equates to approximately 0.5% of the total circulating blood volume. It is not anticipated that the study samples taken will have any adverse effects on clinical outcomes, also considering that eligible patients will be receiving blood transfusions during their acute phase of treatment.

#### 7.4.4 Haematology

Standard haematology measures will comprise Haemoglobin levels (Hb) and Haematocrit (Hct). These will be determined at baseline and 24 hours.

In addition, White Blood Cells count (WBCs count) shall be conducted at baseline and at 24 hours.

#### 7.4.5 Blood chemistry

Standard laboratory assays for blood chemistry shall be conducted to determine Bilirubin, Creatinine, Urea, These will be determined at baseline and 24 hours post admission.

#### 7.4.6 Arterial blood tests

Arterial blood gases to determine pH, oxygen tension (PaO2), carbon dioxide tension (PaCO2), Base Excess, levels of Lactate and $Ca^{2+}$.will be taken:

- At hour 0 (within 10 minutes) immediately following study enrolment
- At haemostasis (within 30 minutes)
- At 6 hours post enrolment (within 30 minutes)
- At 24 $\pm$ 1 hours post enrolment

#### 7.4.7 Conventional Coagulation Tests

Conventional Coagulation Tests (CCT) shall be conducted for all subjects in the CCT arm at each time point detailed in section 7.4.3 up to 24 hours.

The tests shall comprise Platelet Counts (PC), activated Partial Thromboplastin Time (aPTT), Prothrombin Time - International Normalized Ratio (PT/INR) and Clauss Fibrinogen assay.

Prothrombin ratio (PTr) and Clauss Fibrinogen shall be measured for all study subjects at each time point detailed in section 7.4.3 up to 24 hours. During active haemorrhage, samples will be taken for CCT analysis at baseline and after every 4 units of RBC until haemostasis. The results will be used to guide intervention provided any planned intervention based upon the previously analysed sample has been administered to the patient. If a planned intervention has not yet been administered, the sample will be taken and analysed (where resources allow) but will not be used to guide intervention. The first sample taken after an intervention is actually administered will be the next sample used to guide intervention based upon the protocol.

#### 7.4.8 Viscoelastic Haemostatic Assays

Viscoelastic Haemostatic Assays (VHA) will be conducted for all subjects in the VHA arm at each time point detailed in section 7.4.3 up to 24 hours.

During active haemorrhage, samples will be taken for VHA analysis at baseline and after every 4 units of RBC until haemostasis. The results will be used to guide intervention provided any planned intervention based upon the previously analysed sample has been administered to the patient. If a planned intervention has not yet been administered, the sample will be taken and analysed (where resources allow) but will not be used to guide intervention. The first sample taken after an intervention

is actually administered will be the next sample used to guide intervention based upon the protocol.

According to pre-designation, each study centre will only conduct VHA using either Thromboelastography (TEG®) or Rotational Thromboelastometry (ROTEM®) to determine parameters:

- RapidTEG® - ACT, MA and Ly30; TEG® Functional Fibrinogen: MA
- ROTEM® - CT, CA5 and Li30 in ExTEM, and CA5 in FibTEM

### 7.4.9 Outcome Measures

***SOFA score***
For a full description please refer to *Section 12.2*

SOFA score will be registered until discharge from ICU.

***Adverse Reactions***
For a full description please refer to *Section 7.7*

***Blood Products & Procoagulants***
Timings, total number (and doses if appropriate) of different blood products and procoagulants administered both pre-hospital and after admission to the study centre, during resuscitation and after 6 and 24 hours shall be recorded including:

- RBC, FFP/Octaplas, Cryoprecipitate, platelets, whole blood and/or autologous RBC from cell salvage
- Coagulation factor concentrates (PCC, fibrinogen, rFVIIa)
- Tranexamic acid

***Fluids***
Timings (during first 24 hours only) and total volume of different fluids administred both pre-hospital and after admission to the study centre until 24 hours shall be recorded including crystalloids, colloids and hypertonic saline.

***Thromboprophylaxis/prothrombotic medication***
Type of medication administered, timings, dose and indication shall be recorded daily until day 28 with particular attention to duration of treatment (stop date).

***Bleeding episodes***
Qualfying episodes shall be defined by radiological evidence and/or clinical suspicion combined with transfusion requirement after initial haemostasis.

***Ventilator-free days***
Calculated by the subtracting the number of days spent on mechanical ventilation from 28.

***Vasopressor days***
Calculated as the total number of days spent on ionotropic drugs, including for instance noradrenaline, dobutamine, vasopressin.

### *Renal replacement therapy days*
Calculated as the number of days spent on haemodialysis or haemofiltration.

### *ICU days*
The total length of stay in the intensive care unit (ICU). If the patient is in the ICU at any time point during a day, this day will be considered an ICU day.

### *Length of Stay*
Length of stay will be recorded in days, for the total number spent in ITU and in Hospital. If the patient is in the hospital at any time point during a day, this day will be considered a hospital day.

### *Surgical episodes*
Description, timing, duration and reasons for all surgical episodes shall be recorded.

### *Thromboembolic events*
Symptomatic venous thromboembolic events shall be recorded, as confirmed by radiology:

| TYPE OF VENOUS THROMBOEMBOLISM | DIAGNOSIS |
|---|---|
| **Deep venous thrombosis** | Accepted methods of diagnosis include:<br>• compression ultrasound<br>• venography |
| **Pulmonary embolism** | Accepted methods of diagnosis include:<br>• CT pulmonary angiogram (CTPA)<br>• Ventilation-Perfusion scan (V/Q or Q scan as per local guidelines) |

Other thromboembolic events such as myocardial infarction and/or stroke shall be identified by standard clinical diagnostic investigation(s).

### *Patient disposition*
First destination after discharge and disposition at 90-day post admission shall be recorded as either home, rehabilitation facility, nursing home, other hospital or other.

### *Quality of Life*
Subject quality of life shall be assessed using the EuroQoL EQ-5D$^{TM}$ questionnaire, a standardised instrument for use as a measure of health outcome. Quality of life assessment will be conducted in the study centre upon discharge of the subject from hospital and at 90 days post admission.

The in hospital (i.e. discharge) questionnaire will be conducted by research investigators with the patient where possible, but may also be completed with patients personal consultee if necessary. The questionnaire can be completed in less than five minutes. Where the subject has already left hospital, the questionnaire will be posted out with a return stamped addressed envelope. The questionnaire provides instructions for completion of the whole questionnaire, and will be accompanied by a cover note requesting return of the completed form within 5 days.

Patients who have not returned the questionnaire within two weeks of the initial request will be telephoned as a reminder to complete the questionnaire and may be asked to complete it over the phone if necessary. A maximum of three recorded

contact attempts will be made via phone and if these are unsuccessful, no further contact will be made and responses will be marked as not returned.

A further EuroQoL EQ-5D™ questionnaire shall be provided to assess subject quality of life at 90 ± 5days post admission. Confirmation with the local (i.e. hospital care record system) and regional resources (i.e. NHS Health & Social Care Information Centre Spine Services) will ensure only surviving patients receive a questionnaire.

## 7.5 STUDY INTERVENTION

All participating centres currently manage critically bleeding trauma patients according to a standardized Massive Transfusion Protocol (MTP) aiming at a ratio of RBC 1: plasma 1: platelets 1 (1:1:1), typically administering plasma from the start of resuscitation and platelets immediately as they become available.

Corresponding and optimized algorithms based on VHA trigger parameters for the VHA arm and CCT results for the CCT arm respectively, have been developed and will be applied in the enrolled subjects.

The same blood products and procoagulants will be employed in both study arms, with existing standard practice in all participating centres being closely aligned to that of the CCT arm.

Enrolled patients will be block randomized per centre to either study arm:

- CCT: Haemostatic resuscitation, based on a MTP aiming at ratio 1:1:1 of blood components (RBC 1: plasma 1: platelets 1) and CCT to guide further resuscitation with blood products and procoagulant factors.

- VHA: Haemostatic resuscitation, based on a MTP aiming at ratio 1:1:1 of blood components (RBC 1: plasma 1: platelets 1) and VHA-guiding further resuscitation with blood products and procoagulant factors.

### 7.5.1 BLINDING

The trial is to be conducted in an unblinded, randomised controlled manner. It will be clearly evident to both the study site trauma team and its research team into which arm of the trial the subject has been randomised.

Where appropriate and possible, the blind will be maintained for staff involved in data analysis and interpretation.

### 7.5.2 STANDARD CARE

All participating centres currently resuscitate according to a 1:1:1 MTP [12].

Current use of additional diagnostics and therapy such as systematic approach according to ATLS principles, early imaging (e.g. X-rays, FAST, CT), activation criteria for MTP, surgical approach applying damage control principles when indicated, the availability and use of interventional radiology), will not be affected in either of the study groups.

All participating centres apply the same principles of care but variation does exist across centres. However the block randomization of subjects by centre will prevent the trial results from being influenced by any such differences.

An optimized initial MTP based on a 1:1:1 balanced transfusion will be implemented in all centres for approx. 2 months prior to initiation of the RCT and standardized as far as local routines and blood product availability allow.

Corresponding and optimized treatment algorithms based on CCT and VHA monitoring will be applied during the resuscitation of subjects enrolled to the CCT and VHA arms respectively.

### 7.5.3 INITIATION OF STUDY CARE

All participating centres will initiate the management of the study population according to local routines regardless of enrolment in the trial. Trial products will be given as an addition to the 1:1:1 baseline MTP:

**1. Activation of MTP (according to local routines)**

- Clinical signs of haemorrhagic shock and initiate first transfusion

**2. Empiric resuscitation**

- **1:1:1** - 1 RBC : 1 FFP/Octaplas : 1 Platelets
- **TXA** - 1g iv + 1g iv 8 hours infusion, if < 3 hours post injury.
  - If 1g administered prehospital, add 1g iv 8 hours infusion

### 7.5.4 RANDOMISED STUDY CARE (CCT ARM)

The clinical course of subjects randomised to the CCT arm will follow a treatment algorithm utilizing CCT results. The results from each blood sample will be acted upon as soon as they are available.

During active haemorrhage, samples will be taken for CCT analysis at baseline and after every 4 units of RBC until haemostasis. The results will be used to guide intervention provided any planned intervention based upon the previously analysed sample has been administered to the patient. If a planned intervention has not yet been administered, the sample will be taken and analysed (where resources allow) but will not be used to guide intervention. The first sample taken after an intervention is actually administered will be the next sample used to guide intervention based upon the protocol.

***Figure 3a*** **- CCT Arm resuscitation**

# CCT arm Algorithm

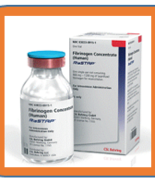

**FIBRINOGEN**
If Fibrinogen <2g/L
Give additional 4g equivalent of fibrinogen
(As Cryoprecipitate or Concentrate)

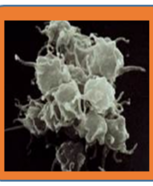

**PLATELETS**
If Platelets <100 x10$^9$/L
Give 1 additional pool of platelets

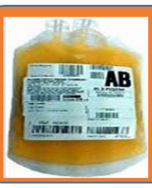


**PLASMA**
If INR >1.2 **AND** Fibrinogen ≥2g/L
Give 4 additional units of plasma

### 7.5.5 RANDOMISED STUDY CARE (VHA ARM)

The clinical course of subjects randomised to the VHA arm will follow a treatment algorithm utilizing VHA results (see *Figure 3b*).

The results from each blood sample will be acted upon as soon as they are available. For the VHA arm, this implies acting upon the parameters as they are appearing, not waiting until the VHA trace is completed.

During active haemorrhage, samples will be taken for VHA analysis at baseline and after every 4 units of RBC until haemostasis. The results will be used to guide intervention provided any planned intervention based upon the previously analysed sample has been administered to the patient. If a planned intervention has not yet been administered, the sample will be taken and analysed (where resources allow) but will not be used to guide intervention. The first sample taken after an intervention is actually administered will be the next sample used to guide intervention based upon the protocol.

A total of 2287 patients were enrolled in the ACIT study [10] during the period 2008-2014. Analyses to define clinically relevant threshold values for VHA interventions were performed on a subset of 2015 patients whose functional coagulation status was recorded using ROTEM, and on a subset of 963 patients studied using TEG®.

Univariate and multivariate statistical modelling with receiver operating characteristic (ROC) curves were used in order to define test performance and identify TEG® parameters significantly associated with coagulopathy (INR>1.2, Fibrinogen < 1g/l, <1,5g/l and <2g/l) and clinical outcomes (i.e. transfusion requirements). Their corresponding threshold values were defined as the most sensitive and specific values using the Youden Index.

In the multivariate model Area Under the Curve (AUC) was calculated for both a multivariate continuous model, a multivariate categorical model and a univariate OR cut off model (when one of the significant values were positive according to cut off value defined by Youden Index).

The univariate and multivariate statistical analyses were performed with R Project for Statistical Computing. The multiple imputations and the rest of the statistical analyses were performed using SPSS Statistics 22. Statistical significance was defined as a p-value < 0.05.

***Figure 3b*** **- VHA Arm resuscitation**

# VHA Algorithm RoTEM ®

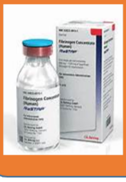

**FIBRINOGEN**
If FIBTEM CA5 < 10mm
Give additional 4g equivalent of fibrinogen
*(As Cryoprecipitate or Concentrate)*

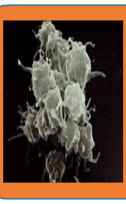

**PLATELETS**
If (EXTEM CA5 - FIBTEM CA5) < 30mm
Give 1 additional pool of platelets

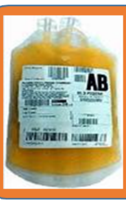


**PLASMA**
If EXTEM CA5 ≥40mm **AND** EXTEM CT >80s
Give 4 additional units of plasma

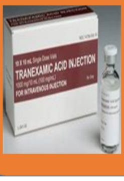


**TRANEXAMIC ACID**
If EXTEM LI30 <85%
Give additional 1g IV bolus of tranexamic acid

# VHA Algorithm rTEG ®

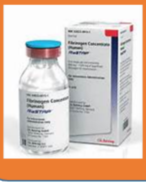

**FIBRINOGEN**
If FF-TEG MA < 20mm
Give additional 4g equivalent of fibrinogen
*(As Cryoprecipitate or Concentrate)*

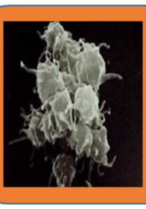

**PLATELETS**
If (RAPID-TEG MA – FF-TEG MA) < 45mm
Give 1 additional pool of platelets

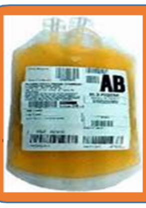


**PLASMA**
If RAPID-TEG MA > 65mm **AND** RAPID-TEG ACT >120
Give 4 additional units of plasma

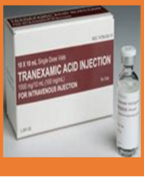


**TRANEXAMIC ACID**
If LY30 >10%
Give additional 1g IV bolus of tranexamic acid

### 7.5.6 CESSATION OF STUDY CARE (HAEMOSTASIS)

For the purposes of this comparative study, the end of intervention shall be defined as the end of resuscitation for active bleeding (i.e. haemostasis).

Hemostasis shall be defined as the point 1 hour from the last administration of RBC and the treating clinician believes primary haemostasis has been achieved.

## 7.6 PROCEDURE FOR DATA COLLECTION

It will be the responsibility of the local researcher(s) to identify eligible adult trauma patients with haemorrhagic shock and ongoing bleeding as soon as possible after the patient has arrived in the ED of the study centre, using local transfusion triggers.

A daily screening log will be completed by research personnel at all participating centres, documenting the total number of adult trauma patients admitted to the ED with major blood loss throughout the recruitment period. Data will be collected for eligible patients who have not been recruited to the study and will be captured on a separate data sheet.

Once final eligibility is confirmed and consent is obtained, the adult trauma patient will be randomised into the study according to the procedures described in Section

7.4. Study subject data will be captured locally using a paper CRF, following local data security routines. Two persons at each centre provide validation of complete and accurate CRF data entry (one has to be a physician). CRF data are transferred and uploaded to a centralised study database whereupon study data integrity is reviewed weekly by the trial coordinating centre.

## 7.7 ADVERSE EVENTS REPORTING

Patients included in this trial have a high risk of morbidity and mortality, with either treatment being administered during a phase of critical bleeding and circulatory failure. Therefore, patients have a very high risk of experiencing several adverse events (AEs) and serious adverse events (SAEs).

The principles of ICH GCP require that both investigators and sponsors follow specific procedures when notifying and reporting adverse events or reactions in clinical trials. These procedures are described in this section of the protocol.

### 7.7.1 Definitions of Serious Adverse Events and Adverse Events

The definitions to be applied to SAEs and AEs recorded in this trial are given in Table 7a below. As this is a trial using blood products and prothrombotic agents, events of interest for safety reporting are those related to transfusion including specifically thromboembolic complications.

**Table 7a: Definitions**

| Term | Definition |
|---|---|
| **Adverse Event (AE)** | Any untoward medical occurrence in a patient or clinical trial subject administered a medicinal product, and which does not necessarily have a causal relationship with this treatment. |
| **Transfusion Related Adverse Reaction or Event** | Any untoward and unintended response to a transfused blood component. |
| **Serious Adverse Event (SAE) or Serious Transfusion related Adverse Reaction** | Respectively any adverse event, adverse transfusion reaction or unexpected adverse transfusion reaction that:<br>• results in death*<br>• is life-threatening**<br>• requires hospitalisation or prolongation of existing hospitalisation***<br>• results in persistent or significant disability or incapacity<br>• Results in a congenital anomaly/birth defect<br>• Other medically significant event |
| **Unexpected Adverse Transfusion Reaction** | An adverse reaction, the nature or severity of which is not consistent with the known reactions to transfusion of a blood component. |

* Death due to the underlying disease or associated conditions will not be reported as an SAE.
**The term ‘life-threatening’ in the definition of ‘serious’ refers to an event in which the patient was at risk of death at the time of the event; it does not refer to an event which hypothetically might have caused death if it were more severe.
***Hospitalisation is defined as an inpatient admission, regardless of length of stay, even if the hospitalisation is a precautionary measure for continued observation. Hospitalisations for a pre-existing condition (including elective procedures that have not worsened) do not constitute an SAE.

- An ***unexpected SAE*** is defined as any event meeting the definition of an SAE above and that is not listed in the protocol as an "expected SAE not subject to expedited reporting" (see 7.7.2 and 7.7.3).

- An ***unexpected related SAE*** is defined as an unexpected SAE that is judged to be possibly, probably or definitely related to the transfusion policy being used in this hospital

### 7.7.2 Expected SAEs not subject to Expedited Reporting (expected occurrences)

Participants in this study are presenting with major blood loss following severe injury. Many of these patients will be expected to develop SAEs during the course of their hospital admission. All SAEs, expected or not, will be recorded on an SAE form. The following situations that fulfil the definition of an expected SAE (7.7.1) are not subject to expedited reporting by the site.

### 7.7.3 Expected SAEs which are Clinical outcome measures

- Deaths
- Bleeding
- Any element of thromboembolic and ischaemic events- (myocardial infarction, stroke, pulmonary embolus, DVT)
- Organ failure (single or multi-)
- Acute lung injury
- Respiratory compromise and need for ventilation and intubation

### 7.7.4 Expected SAEs which are not Clinical outcome measures

- Infection
- Congestive cardiac failure
- Respiratory complication:
    - Aspiration pneumonia
    - Pulmonary oedema
- Transient ischaemic attack
- Acute transfusion reactions (see section 12.5.1)
- Non-acute transfusion reactions (see section 12.5.2)

**Note: Any event meeting the definition of an SAE and not listed above will be treated as an "unexpected SAE".**

### 7.7.5 Notification and Reporting of Serious Adverse Events

Serious Adverse Event (SAEs) that are considered to be 'related' and 'unexpected' are to be reported to the sponsor within 24 hours of learning of the event and to the Main REC within 15 days in line with the required timeframe.

### 7.7.6 Urgent Safety Measures

The CI may take urgent safety measures to ensure the safety and protection of the clinical trial subjects from any immediate hazard to their health and safety. The measures should be taken immediately. In this instance, the approval of the REC prior to implementing these safety measures is not required. However, it is the responsibility

of the CI to inform the sponsor and Main Research Ethics Committee (via telephone) of this event immediately.

The CI has an obligation to inform both the Main REC in writing within 3 days, in the form of a substantial amendment. The sponsor (Joint Research Management Office [JRMO]) must be sent a copy of the correspondence with regards to this matter.

#### 7.7.7 Investigator Responsibilities

The Chief Investigator has overall responsibility for the conduct of the study. As this is a multi-site study, the Principal Investigator has responsibility for the research at their local site and is responsible for informing the Sponsor of all reportable SAEs that occur at their site following the guidelines below.

- The Sponsor should be notified within 24 hours of the Principal Investigator becoming aware of the SAEs and outcomes listed in section 7.3. Investigators should notify the Sponsor of all such events occurring up to study Day 28.
- The relevant forms must be completed by the Investigator (the consultant named on the delegation of responsibilities log who is responsible for the patient's care). In the absence of the Investigator, the form should be completed and signed by a member of the site trial team and faxed or emailed. The responsible Investigator should subsequently check, annotate and sign the form and re-fax/email to the Sponsor as soon as possible. The initial report must be followed by detailed written reports as appropriate.
- The investigator must follow-up all reported SAEs and clinical outcomes which require expedited reporting (whether they are expected or not) until resolution or the event is considered stable.
- Investigator must supply the Sponsor, REC and relevant NHS Trust R&D with any supplementary information they request.

### 7.7.8 Annual Safety Reporting

The CI will send the Annual Progress Report to the main REC using the NRES template (the anniversary date is the date on the MREC "favourable opinion" letter from the MREC) and to the sponsor.

### 7.7.9 Statutory Reporting

Hospital staff remain responsible for reporting all transfusion-related adverse events to SHOT/SABRE according to standard procedures, as required under the regulations of the EU Blood Directive. Staff at the institution are also responsible for notifying their local R&D department of SAEs (as per the institutions standard local procedure).

**SAE REPORTING**

Within 24 hours of becoming aware of a reportable SAE please fax a completed SAE form to the Sponsor on:

+44 20 7882 7276

Or E-mail information to: research.safety@bartshealth.nhs.uk

## 7.8 INVESTIGATORS ASSESSMENT

### 7.8.1 Seriousness

The Chief/Principal Investigator responsible for the care of the subject, or in his absence an authorised medic within the research team, is responsible for assessing whether the event is serious according to the definitions given in *Section 7.7.1.*

### 7.8.2 Causality

The Investigator must assess the causality of all serious adverse events/reactions in relation to the trial treatment according to the definition given. If the SAE is assessed as having a reasonable causal relationship, then it is defined as a SAR.

### 7.8.3 Definitions of Causality

| Relationship | Description |
|---|---|
| **Unrelated** | There is no evidence of any causal relationship |
| **Unlikely** | There is little evidence to suggest there is a causal relationship (e.g. the event did not occur within a reasonable time after administration of the trial drug or intervention). There is another reasonable explanation for the event (e.g. the patient's clinical condition, other concomitant treatment). |
| **Possible** | There is some evidence to suggest a causal relationship (e.g. because the event occurs within a reasonable time after administration of the trial drug or intervention). However, the influence of other factors may have contributed to the event (e.g. the patient's clinical condition, other concomitant treatments). |
| **Probable** | The evidence is clearly in favor of attributing the adverse reaction to the trial drug or intervention |
| **Definitely** | There is conclusive evidence beyond reasonable doubt attributing the adverse reaction to the trial drug or intervention. |
| **Not assessable** | There is insufficient or incomplete evidence to make a clinical judgment of the causal relationship. |

#### 7.8.4 Expectedness

The investigator must assess the expectedness of all SARs according to the definition given. If the SAR is unexpected, then it is a SUSAR.

### 7.9 SAFETY ANALYSIS

A pre-defined interim analysis will be performed after the enrolment of 100 patients, including an assessment of recruitment logistics with the possibility to revise the planned sample size.

A Data Safety Monitoring Board (DSMB) will review all data on outcome of the patients in the respective treatment arms. The DSMB will focus on adherence to protocol, and present pre-specified criteria that need to be fulfilled with regard to safety of the patients for the study to continue.

### 7.10 SUBJECT WITHDRAWAL

Every reasonable effort will be made to maintain protocol compliance and to retain patient participation in the study, consistent with the provisions of informed consent and good clinical practice. The following are potential reasons why a patient may be withdrawn from the study:

1. Withdrawal of consent/agreement: the patient, the patient's personal consultee, independent physician, or designated individual who had provided initial consent/agreement to enter the study may withdraw consent/agreement at any time throughout the duration of the trial, without prejudice to future medical care and treatment.
2. Retrospective exclusion: If a patient is deemed to not meet one or more of the inclusion/exclusion criteria in retrospect they will be withdrawn from the study.
3. Major protocol deviation from the study design by the subject, observed or suspected by the investigator.
4. Administrative: the sponsor or monitoring committees decide to terminate or discontinue the study.
5. The subject's health would be jeopardised by continued participation and is withdrawn at the discretion of the investigator.

The study withdrawal form will be completed for these patients and a reason for withdrawal captured. All subject's withdrawn from the study will be managed in accordance with the hospital's standard procedures.

### 7.11 DATA COLLECTION AND FOLLOW-UP FOR WITHDRAWN SUBJECTS

Patients who withdraw from the study after randomisation should be followed for safety by conducting safety assessments through to the end of day 28. If a patient who withdraws has an ongoing SAE every effort must be made to follow such events until satisfactory resolution is obtained or until further follow-up is no longer warranted.

### 7.12 SUBJECT REPLACEMENT

Subjects who withdraw from the study will be replaced.

## SCHEDULE OF INTERVENTION (in Diagrammatic Format)

| ASSESSMENT | SCREENING & RANDOMISATION | RESUSCITATION (Every 4U RBC) | HAEMOSTASIS (1hr after last RBC) | 6 HRS | 24 HRS | UNTIL DAY 28 (Daily) | DISCHARGE | DAY 90 |
|---|---|---|---|---|---|---|---|---|
| **Eligibility** | X | | | | | | | |
| **Informed Consent** | X | | | | | | | |
| **Medical History** | X | | | | | | | |
| **Physical Exam** | X | X | X | X | X | | | |
| **Prior Surgery & Treatment** | X | | | | | | | |
| **Blood Sample** | X | X | X | X | X | | | |
| **Haematology** | X | | | | X | | | |
| **Blood chemistry** | X | | | | X | | | |
| **Arterial Blood Tests** | X | | X | X | X | | | |
| **Conventional Coagulation Tests (CCT)** | X | X | X | X | X | | | |
| **Visco Haemostatic Assays (VHA)** | X | X | X | X | X | | | |
| **Total Blood, Coag Factor, Fluid & TXA** | X | X | X | X | X | | | |
| **SOFA** | X | | | | X | end ICU | X | |
| **SAEs** | X | X | X | X | X | X | X | |
| **Mortality** | | | | X | X | X | | X |
| **Ventiliator-free days** | | | | | | X | X | |
| **Vasopressors days** | | | | | | X | X | |
| **Renal Replacement Therapy days** | | | | | | X | X | |
| **ICU days** | | | | | | X | X | |
| **Hospital days** | | | | | | X | X | |

| | | | | | | | | |
|---|---|---|---|---|---|---|---|---|
| **Surgical episodes** | | | | | | × | × | |
| **Thromboembolic events** | | | | | | × | × | |
| **First destination** | | | | | | | × | |
| **Quality of Life** | | | | | | | × | × |
| **Current disposition** | | | | | | | | × |

### 7.13 END OF STUDY DEFINITION

The study will be considered closed when all surviving Subjects complete in-hospital safety and outcomes monitoring. This includes: safety measures of serious adverse reaction rate within 28 days, total hospital stay, total critical care stay, 28-day ventilator free days, and 28-day mortality.

## 8 STATISTICAL CONSIDERATIONS

### 8.1 SAMPLE SIZE

The planned sample size for this study is 392 patients for which MTP is activated and transfusions initiated, 196 in each study arm.

Based upon legacy registry data from the Partners, approximately 28% of patients will need massive transfusion or die. This figure decreases to an overall proportion of 15% in the VHA group (i.e. using VHA guided strategy). With a power of 80% and a two-sided alpha of 0.05, 170 patients per group are required. Assuming a drop-out rate of 15% 196 patients are needed per group.

### 8.2 METHOD OF ANALYSIS

**Subject characteristics**
Continuous patient characteristics will be described as means with standard deviation or median, with interquartile range according to normal distribution. Normality will be checked using the histograms and Kolmogorov-Smirnoff test. Categorical patient characteristics will be described as number of patients and percentages.

Differences between centres will be explored and if detected, all analyses will also be corrected for centre using either a logistic regression or linear regression.

**Endpoints**
The primary endpoint of massive transfusion and death will be assessed by difference in proportion, using the Chi-square test or Fisher exact test as appropriate. Absolute risk reductions and relative risk reductions by VHA-guided therapy will be calculated. A two-sided p-value<0.05 will be considered significant.

Differences in secondary endpoints will be assessed by difference in means and tested using the Students-t test, or difference in median and tested using the Mann-Whitney U test as appropriate.

Calculating the area under the curve will assess the duration of and severity of coagulopathy until haemostasis. Differences between the two treatment arms will then be examined using Students t-test. Both ROTEM®-guided and TEG®-guided therapy together (i.e. VHA arm) will be compared with the CCT arm, as well as separate analyses for ROTEM®-guided and TEG®-guided therapy alone in regard the primary endpoints and correction of coagulopathy.

All analyses will be based on an intention to treat analysis, however a per-protocol analysis and a sensitivity analysis will also be performed for the primary endpoint. The following patients will be excluded from the per protocol analysis:

- Patients who do not have at least one ROTEM®/TEG®/CCT test performed
- Patients who die within 60 minutes after baseline blood sampling
- Patients who achieve haemostasis within 60 minutes of baseline sampling.

**Subgroup analyses**

The following patient subgroups will also be analysed separately:

- Patients with severe traumatic brain injury (defined as brain abbreviated injury score ≥4)
- Patients without severe traumatic brain injury (brain abbreviated injury score <4)
- Patients with known pre-existing coagulopathy
- Prior oral anticoagulant therapy except for aspirin

**Integrated cost-effectiveness analysis**

A cost-effectiveness analysis will be conducted to assess the costs and effects of VHA-guided therapy versus those of optimised empiric treatment. A model will be developed which will be structured around the key clinical time points and events in the early management pathway of bleeding trauma patients, for example discrete time periods modelled might include 0-3 hours post presentation, 3-6 hours, 6-12 hours and 12-24 hours with outcomes at the end of each period comprising: patient alive and free of massive transfusion, patient alive and massive transfusion ongoing, and patient deceased.

For each country individually, data from the CCT arm of the trial on numbers of patients experiencing the outcomes at the end of each period (together with the treatments (and their associated costs) received by patients in each time period) will be used to populate the model. Country-specific trial data (supplemented with trauma registry data) on survival and costs from 24 hours to 28 days (plus HRQoL at 28 days) will be added, and for patients still alive at 28 days, longer-term quality of life, life expectancy, and costs will be modelled using a combination of data from the trauma registry and the published literature).

In an identically structured VHA arm of the model, estimates of the trial's overall treatment effect (relative risk reduction) with VHA guided therapy will be used to adjust the outcomes in the CCT arm of the model for each country, so as to estimate the potential additional proportions of patients alive and free from massive transfusion at 24 hours with VHA testing.

The additional costs associated with VHA will be added to this arm of the model, together with the costs of treatment received by VHA trial patients in each of the discrete time periods to 24 hours. As per the CCT arm, country-specific trial data

(supplemented with trauma registry data) on survival and costs from 24 hours to 28 days (plus HRQoL at 28 days) will be added, and for patients still alive at 28 days, longer-term quality of life, life expectancy, and costs will be modelled using a combination of data from the trauma registry and the published literature).

The two treatment policies will be compared in terms of their estimated costs and effects (quality adjusted life years (QALYs): calculated by combining survival and HRQoL data) and incremental analyses will be performed. If VHA-guided therapy is more effective but also more costly than empirical treatment then the incremental cost-effectiveness ratio (ICER) will be calculated. The ICER is calculated by dividing the difference in costs between VHA and empirically guided therapy by the difference in effects (QALYs) and gives the additional cost of generating one additional unit of outcome (here a QALY).

So as to account for the uncertainty in the model input data, parameters will be entered as distributions rather than point estimates. Probabilistic sensitivity analysis will be used to take repeated random draws from all distributions simultaneously, each time recalculating the model's results for a total of 2000 times. The uncertainty will be summarised on the cost-effectiveness plane and using cost-effectiveness acceptability curves. For each country the modelling exercise should provide an estimate of the probability that VHA-guided therapy is likely to be cost-effective when compared with optimised empiric treatment.

# 9 ETHICS

## 9.1 SAFETY CONSIDERATIONS

Conducting clinical studies in trauma is challenging, with important safety considerations and needs concerning the priority for delivering life-saving patient care, whilst achieving the robust enrolment of subjects in the emergency setting.

This multicentre study will be achieved by employing a flexible trial protocol that accommodates variance in practice across sites and meets resource demands such that:

- The study will be conducted by dedicated research personnel, without obligations to the actual treatment of the subject, thereby ensuring both focused patient care and compliance to the study protocol
- Comprehensive screening and the conduct of study procedures will be achieved by the availability of multiple skilled research personnel, recruiting subjects presenting *in extremis* and often out of normal working hours (i.e. throughout the night).

## 9.2 RISKS

All parts of the study will be carried out to avoid patient risk and minimize discomfort at all times. At no time will patient care be compromised or delayed for the purposes of the study.

The study comprises a VHA arm that utilises a patient-matched treatment algorithm developed from the analysis of data from over 2,000 trauma patients describing haemostatic impact of different types of trauma-induced coagulaopthy and therapeutic interventions with blood products and pro-coagulants. The algorithms aim

to normalize haemostatic competence, as evaluated by functional haemostatic assays such as TEG® and ROTEM®, since all published evidence to date suggest that normal haemostatic competence in bleeding trauma patients is associated with improved outcome.

This is a randomised controlled trial in which all subjects regardless of randomisation will initially receive care encompassing a MTP aiming for a 1:1:1 ratio of RBC: plasma: platelets, according to the best currently available evidence [12]. Half of the patients (Intervention group) will be allocated to receive patient-matched VHA algorithm-led resuscitation in addition to the empiric MTP. The other participants (CCT group) will have their resuscitation adjusted in response to standard clinical and laboratory results (CCT).

There is a clear rationale and requirement for sequential blood sampling of study subjects during their acute phase of clinical care within the ED. The status and response of the patient's functional coagulation during resuscitation is a dynamic process and the full picture will not be apparent on a single baseline blood draw. Many trauma patients exhibit a coagulopathic state either at admission or in the later stages of the body's response to injury or treatment itself. How these changes manifest is poorly described, but would be defined by this protocol.  This would have major implications for the future diagnosis and treatment of coagulopathy.

As most major trauma patients have an arterial or central line placed, most blood draws are not painful to the patient. Whenever possible the investigators will coordinate study blood draws with those of clinical need, to minimise disturbing the patient and the number of needle-sticks. The risks of blood sampling are limited to some potential bruising at the site of venepuncture, and discomfort to needle puncture (where no arterial line is already in place).

All serious adverse events associated with the study will be reviewed as they occur (see *Section 7.7*). Additionally, a pre-defined interim analysis will be performed after the enrolment of 100 patients in which all safety data of the patients in the respective treatment arms will be reviewed by a DSMB.

Participation in research may involve some degree of loss of privacy. However this risk will be minimized by the data protection methodology (see *Section 10*), although the study does not include any tests that might subsequently result in significant personal, financial or social risk to research subjects.  Every effort will be made to ensure that the study data is secured and patients' privacy remains protected.

## 9.3 BENEFIT TO THE PATIENT

There may be a direct benefit to those subjects receiving the Intervention.

There is no incentive for participation in the study.

## 9.4 BENEFIT TO SOCIETY

Trauma remains the leading cause of death and disability in patients under 45 years of age. Trauma patients tend to be young, active members of society, often with good jobs and young families to support. The outcome of trauma patients is determined in the first few hours following injury. Although the study may not carry a direct benefit to all its subjects, a societal benefit lies in the expected results of the trial that will

deliver evidence-based guidelines for the identification and management of coagulopathic bleeding in the trauma population.

## 9.5 PATIENT ENROLMENT

Eligible subjects will be enrolled at specialist trauma-receiving hospitals located in Amsterdam, Cologne, Copenhagen, London, Oslo and Oxford. Each study centre shall submit ethical application to their respective ethics committees for research on human subjects.

### 9.5.1 INFORMED CONSENT PROCEDURES

Most, but potentially not all, subjects will be incapacitated at the time of eligibility (critical injury, mechanical ventilation, sedation), such that the Mental Capacity Act (England; 2005) and Declaration of Helsinki (World Medical Association; 2013) provides guidance.

Patients will be recruited within 3 hours of their injury and within 1 hour of admission to the ED of the study site. This is at a particularly stressful time for relatives and families, an important consideration when the intervention under investigation is time sensitive. This study requires that the intervention be given rapidly, thereby necessitating that eligible patients are consented very soon after hospital admission. As injury is an unexpected event, it is uncommon that relatives are present at the time of hospital admission.

As stipulated by International Conference on Harmonisation guidelines for Good Clinical Practice the subject and/or their Personal Consultee (PC) (e.g. next of kin) should be given ample time to consider giving their consent/agreement for the study. It is felt that 24 hours gives sufficient time for the patient and/or PC to consider participation within the study and give informed consent/agreement. However, the need for urgent treatment in this trial means that the implementation of the research cannot be delayed and that it would be inappropriate to delay treatment until fully informed consent/agreement can be obtained from the patient, relative or other PC. Patients who are incapable of giving consent in emergency situations are an established exception to the general rule of informed consent in clinical trials.

As the timeframe required for subject or PC consent/agreement is not compatible with the time sensitivity of this trial, several approaches to obtaining informed consent/agreement will be used, all of which are consistent with the Mental Capacity Act (England; 2005) and the Declaration of Helsinki (2013):

Declaration for initial enrolment in the trial will be sought from a Nominated Consultee (NC), in the form of independent clinicians (e.g. trauma team leader) who are familiar with this study's consenting process and are present at the trauma call. Senior Emergency Department clinicians acting as NC will receive information about the study by a combination of an oral presentation and a written information sheet and have the opportunity to ask questions and discuss the study with investigators prior to study enrolment.

If PCs are present, bearing in mind the clinical situation and their level of distress, they will be provided with brief information about the trial either verbally or in writing. Specifically, the investigator will explain to the PC that the patient will receive the usual emergency treatments for traumatic haemorrhage but that in addition to these,

the patient has been enrolled in a research study that aims to improve the outcome of patients with this condition.

It will be explained that the study is being conducted to see whether using a diagnostic test to guide personalised transfusion therapy will improve patients' outcome by stemming bleeding more quickly. The relative will be informed that the patient will be treated by transfusion of the same blood products and clotting agents, initially by a massive transfusion protocol aiming to deliver blood products in fixed ratio and subsequently in a pre-determined manner according to test results obtained during the bleeding patient's treatment, either conventional clotting tests (CCT arm) or more sophisticated coagulation monitors (VHA arm). The investigator will explain that whilst we hope that use of the diagnostic test will improve outcome after trauma haemorrhage, we cannot presently be sure of this.

If the PC objects their wishes will be respected. If no PC is present, two doctors (one independent of the trial) will consider the patient's eligibility criteria and any known views of the patient about trial participation. Together they will decide whether or not to enrol the patient into the trial.

We do not propose to include a telephone contact with relatives/personal consultees in order to minimise stress and anxiety at a difficult time.

If and when subjects regain the physical and mental capacity to give consent, information will be provided to them and written informed consent will be sought for continuation in the trial. If a patient or representative declines to give consent/agreement for continuation at this stage, his/her wishes will be respected. For any patient who was included but did not regain full capacity, agreement will be sought from a relative or other appropriate representative for continuation of the trial. In this case, an attempt will be made by the investigator to discuss the trial with the PC during the daily visit for data collection if they are present and it is deemed an appropriate time for the discussion to take place. These attempts will continue until PC or subject consent/agreement is obtained. All interactions and attempts at contact with the PC and/or subject will be documented in a study consent log.

Patients who die during the follow up period or do not regain mental capacity, will be included in the study based on the advice provided by the Personal Consultee and/or nominated consultee. In cases where the subject dies before we have had the opportunity to discuss the trial and obtain consent/agreement from the patient or the Personal Consultee, the patient will remain in the trial based on the signed declaration obtained by the Trauma Team Leader (nominated consultee) and we will not attempt future telephone or written contact with relatives/Personal Consultee in order to minimise stress and anxiety associated with the unexpected and traumatic death of their relative / next of kin. Should an investigator have had the opportunity to introduce themselves and discuss the trial with the PC prior to the subject's death but written agreement has not yet been obtained, then we will make a maximum of three further attempts (by any combination of telephone, e-mail or letter) as deemed appropriate in each individual case to contact the PC and obtain written agreement. If after these further attempts, the PC has either not been contactable or has not returned written agreement, then the subject shall remain in the trial based on the NC declaration. If the PC objects to the subject remaining in the study at this stage then their wishes will be respected.

In summary, we believe these approaches are justified under the conditions of the Mental Capacity Act (England; 2005) and Declaration of Helsinki (World Medical Association; 2013) for the following reasons:

- The urgent/emergency nature of the intervention

- The proposed recruiting centre has used this consenting procedure for previous randomised controlled trials in trauma (MP4OX Ph2b - EudraCT: 2010-023129-39; Cryostat Ph2a – ISRCTN55509212) and the ongoing ACIT-2 observational study (REC ref: 07Q0603/29) that has successfully recruited more than 1300 patients to date.

Prior to any study related activities and subsequently for continuation when the subject is capable or from a relative or representative, two copies of the Informed Consent/agreement Form approved by the Research Ethics Committee must be signed and dated. One original copy must be retained by the site in its study file, together with any subsequent approved amended versions. The other original must be given to the subject for his or her own records.

### 9.5.2 REMOVAL OF PATIENTS FROM THE STUDY

The patient, the patient's personal consultee, independent physician, or designated individual who had provided initial consent or approval to enter the study may withdraw consent/agreement at any time throughout the duration of the trial, without prejudice to future medical care and treatment of the patient.

## 10 DATA HANDLING AND RECORD KEEPING

### 10.1 CONFIDENTIALITY

The Investigator has responsibility to ensure patient anonymity is protected and maintained. They will also ensure their identities are protected from any unauthorised parties. Information regarding study patients will be kept confidential and managed in accordance with the Data Protection Act, NHS Caldicott Guardian, The Research Governance Framework for Health and Social Care and Research Ethics Committee Approval.

The Investigator as well as the study team will adhere to these parameters to ensure the subject's identity is protected at every stage of participation within the study. To ensure this is done accordingly, each patient, at time of consent will be allocated a unique screening number by either the PI or a member of the study team before undergoing any screening procedures. The subject's initials (the first letter of their first name and the first letter of their last name) will be used as a means of pseudo-anoymising parameters. This information will be kept on a screening log, and updated accordingly throughout the study. Once the patient has completed screening procedures and is enrolled onto the study, they will be allocated a unique randomisation number by the investigator (see *Section 7.2*).

Subject identifiable information (name, date of birth, hospital number) will be recorded for the purposes of consent and data collection including AEs and SAEs (hardcopy Case Report Form). In addition, contact information for the subject (address, telephone number and General Practitioner Details) will be recorded in the consent log in the event an investigator may need to contact the subject or their GP

with regards to the trial. Access to all identifiable information will be limited to the study investigators.

If any subject information needs to be sent to a third party (including correspondence/communication to central laboratories, CROs, sponsor) the PI and the study team will adhere to patient pseudo-anonymous parameters. This includes the patient initials, date of birth, gender as well as the unique study ID/randomisation number. Any information that is to be collected by these third parties will utilise these coded details for any relevant documents as well as maintaining databases.

All Investigators agree that all information communicated by the sponsor is the exclusive property of the sponsor and will ensure that the same shall be kept strictly confidential or any other person connected with the work and shall not be disclosed to any third party without the prior written consent of the sponsor.

All rights and interests worldwide in any inventions, know-how or other intellectual or industrial property rights which arise during the course of and/or as a result of the clinical study which is the subject of the protocol or which otherwise arise from the information or materials supplied under this agreement, shall be assigned to, vest in and remain the property of the sponsor.

## 10.2 STUDY DOCUMENTS

- A signed protocol and any subsequent amendments
- Current Summary of Product Characteristics/ Investigator's Brochure
- Sponsor Self-Monitoring template for the trial team to complete on a regular basis as detailed by the Monitoring section
- Current/Superseded Patient Information Sheets
- Current/Superseded Consent Forms
- Indemnity documentation from sponsor
- Conditions of Sponsorship from sponsor
- Conditional/Final R&D Approval
- Signed site agreement
- Ethics//approvals/correspondence
- CVs of CI and site staff
- UK regulations (GCP) course certificate of each of trial team
- Laboratory accreditation letter, certification and normal ranges for all laboratories to be utilised in the study
- Delegation log
- Staff training log
- Site signature log
- Patient identification log
- Screening log
- Enrolment log
- Monitoring visit log
- Protocol training log
- Correspondence relating to the trial
- Communication Plan between the CI/PI and members of the study team
- SAR reporting plan for the study

## 10.3 CASE REPORT FORM

Data collection for this study will be accomplished using a paper case report form (CRF) to capture data prospectively and transferred to an electronic data capture system. CRFs are required and will be completed for each randomized subject. It is the Investigator's responsibility to ensure the accuracy, completeness and timeliness of the data reported on the subjects CRF. CRFs will be completed in a timely fashion to support the study timelines. Source documentation supporting the CRF data will indicate the subject's participation in the study and document the dates and details of informed consent/agreement and study procedures. Data collected on the CRF will be verified against the source documentation.

A Summary of Data Capture for the CRF is shown below:

- Eligibility/exclusion criteria checklist
- Informed consent/agreement including dates
- Demographics (including age, gender & hospital number)
- Date of screening
- Randomisation code
- Medical history (including pregnancy, pre-injury anticoagulant therapy or coagulopathy)
- Physical examination (including weight, vital signs & injury severity)
- Blood products and fluids administered over first 24 hours
- Conventional laboratory & VHA test results
- Surgical procedures (including duration)
- Thromboprophylactic medication
- SAE (see *Section 7.7*)
- Mortality (including time to death)
- SOFA score from admission to day 28 or discharge (see *Section 12.2*)
- Outcomes at discharge or day 28 (including total length of hospital stay, critical care stay, renal replacement therapy & ventilator-free days)
- 90-day mortality status

## 10.4 IDENITIFCATION OF SOURCE DATA

Source data includes all information in original records and certified copies of original records of clinical findings, observations or other activities in a clinical trial necessary for the reconstruction and evaluation of the trial. Source data are contained in source documents (original records or certified copies).

The following items are defined as source data:

- Subject medical notes including prescription and observation charts.
- Barts Health NHS (Royal London Hospital site) electronic Care Records Service for access to clinical laboratory test results, radiology general practitioner or subject contact information.
- Subject monitoring equipment (vital signs).
- NHS Spine database for confirmation of 90 day mortality status.
- Correspondence (telephone or writing) with Subject.

Data such as weight, vital signs, heart rate, temperature, pregnancy test will be recorded in subject notes or on a study specific source data sheet. Where the CRF is used as source document for this trial, the data to which this applies will be documented in a File Note.

## 10.5 RECORD RETENTION AND ARCHIVING

It is the responsibility of the Chief Investigator (CI) to maintain adequate records for the study including completed CRFs, signed Informed consent/agreement documents, drug disposition records and all correspondence with the REC and the sponsor.

The CI must make study data accessible to the monitor andother authorised representatives of the Sponsor upon request. A file for each subject must be maintained that includes the signed Informed Consent/agreement form and the Investigator's copies of all source documentation related to that subject. The Investigator must ensure the reliability and availability of source documents from which the information on the CRF was derived.

All records are to be retained in a secure location for a minimum period of 20 years after the study has completed, as per the Research Governance Framework and Trust Policy. For trials involving BHT patients, undertaken by Trust staff, or sponsored by BHT or QMUL, the approved repository for long-term storage of local records is the Trust Modern Records Centre, which is based at 9 Prescot Street.

## 10.6 COMPLIANCE

The CI will ensure that the trial is conducted in compliance with the principles of the Declaration of Helsinki (2013), Research Governance Framework for Health & Social Care (2005), Principles of ICH-GCP, European Commission Directives 2001/20/EC and 2005/28/EC with the implementation in national legislation in the UK by Statutory Instrument 2004/1031 and any subsequent amendments, Trust and Research Office policies and procedures and any subsequent amendments.

## 10.7 CLINICAL GOVERNANCE ISSUES

### 10.7.1 Ethical considerations

This protocol and any subsequent amendments, along with any accompanying material provided to the subject in addition to any advertising material will be submitted by the Investigator to an Independent Research Ethics Committee. Written Approval from the Committee will be obtained and subsequently submitted to the JRMO to obtain Final R&D approval.

Verification of the Ethics Committee's unconditional approval of the protocol, subject information sheet and informed consent document will be transmitted to the sponsor prior to shipment of clinical supplies.

The written, unconditional approval from the Ethics Committee must refer to the study by exact protocol title and number, identify the documents reviewed and state the date of review. Any amendment to the protocol must be approved in the same way.

The ethics committee must be informed by the investigators of all subsequent protocol amendments and of serious or unexpected adverse experiences occurring during the study, which are likely to affect the safety of the subjects or the conduct of the study.

### 10.7.2 Regulatory Approval

The sponsor will be responsible for obtaining all relevant approvals to conduct the study, in accordance with any applicable requirements, prior to a site initiating the study and prior to any subsequent amendments.

### 10.7.3 Amendments

The study will be conducted according to this protocol. Any changes in procedure can only be implemented on completion of an amendment, the only exception to this will be if it is necessary to make a change due to urgent safety measures to protect the safety, rights or welfare of subjects.

Protocol amendments must be made only with the prior approval of the sponsor. Amendments will be classified as non-substantial or substantial by the sponsor.

In accordance with the Medicines for Human Use (Clinical Trials) Regulations (2004), non-substantial amendments can be made at any time during the trial with the prior approval of the sponsor. These will be documented and recorded as relevant.

For a substantial amendment to the protocol or supporting documents the sponsor will obtain approval as relevant from the REC.

The substantial amendments will also be notified to the JRMO.

### 10.7.4 Subject Compensation for Adverse Effects on Health

The sponsor will adhere to local regulations regarding Clinical Trial Compensation Guidelines to subjects whose health is adversely affected by taking part in the study.

# 11 QUALITY CONTROL AND QUALITY ASSURANCE

## 11.1 SUMMARY MONITORING PLAN

The site Clinical Trials Coordinator will perform regular monitoring of trial documentation and CRFs. During the set-up visit investigators will be given all relevant documentation and training in order for them to participate safely and effectively in the trial. The first monitoring visit will take place after the first subject is randomized into the trial. The monitoring plan will include 100% monitoring of consent/agreement forms and source data verification on a proportion of CRFs (including 100% verification of the data collected from the first 10 patients recruited). Investigators/institutions are required to provide direct access to source data/documents for trial-related monitoring, audits, ethics committee review and regulatory inspection.

## 11.2 AUDIT AND INSPECTION

For the purpose of compliance with Good Clinical Practice (GCP) and Regulatory Agency Guidelines it may be necessary for the sponsor or a Drug Regulatory Agency to conduct a site audit. This may occur at any time from the start to after conclusion of the study. A study may be identified for audit by any method listed below:

- A project may be identified via the risk assessment process.
- An individual investigator or department may request an audit.
- A project may be identified via an allegation of research misconduct or fraud or a suspected breach of regulations.
- Projects may be selected at random. The Department of Health states that Trusts should be auditing a minimum of 10% of all research projects.
- Projects may be randomly selected for audit by an external organisation.

Internal audits will be conducted by a sponsor's representative.

When an Investigator signs the protocol, he/she agrees to allow the Drug Regulatory Agency or the sponsor auditors to inspect his/her study records. Furthermore, if an Investigator refuses an inspection, data from that centre will not be accepted in support of a New Drug Registration and/or Application.

## 11.3 SERIOUS BREACHES IN GCP OR TRIAL PROTOCOL

The sponsor of the Clinical Trial is responsible for notifying the licensing authority in writing of any serious breach of:

- The conditions and principles of GCP in connection with that trial; or
- The protocol relating to the trial, as amended from time to time in accordance with regulations 22 to 25, within 7 days of becoming aware of that breach.

For the purposes of this regulation, a 'serious breach', is a breach which is likely to effect to a significant degree:

- The safety or physical or mental integrity of the subjects of the trials; or
- The scientific value of the trial.

The CI is responsible for reporting any serious breaches to the sponsor (JRMO) **within 24 hours**.

## 11.4 NON-COMPLIANCE

Non-compliance is a noted systematic lack of both the CI and the study staff adhering to SOPs/protocol/ICH-GCP and UK regulations, which leads to prolonged collection of deviations, breaches or suspected fraud.

Instances of non-compliance may be captured from a variety of different sources including monitoring visits, CRFs, communications and updates. The sponsor will maintain a log of the non-compliances to ascertain if there are any trends developing which to be escalated. The sponsor will assess the non-compliances and action a

timeframe in which they need to be dealt with. Each action will be given a different timeframe dependent on the severity. If the actions are not dealt with accordingly, the JRMO will agree an appropriate action, including an on-site audit.

### 11.5 SPONSORS TERMINATION OF STUDY

The sponsor reserves the right to discontinue the clinical study at any time for medical or administrative reasons. When feasible, a 30-day written notification will be tendered.

### 11.6 INDEMNITY AND INSURANCE

The sponsor will provide indemnity in accordance with the agreement with the trial centre. The investigator is required to have adequate current insurance to cover claims for negligence and/or malpractice. The sponsor will provide insurance cover for the clinical trial as required by national regulations.

### 11.7 POST-TRIAL CARE

The subjects will remain in the study until they are discharged from the hospital. Consequently there is no requirement for post-trial care.

# 12 APPENDICES

## 12.1 REFERENCES

**1:** Mock C. Strengthening Care for the Injured Globally. *The Journal of Trauma: Injury, Infection, and Critical Care.* 2011;70(6):1307-1316.

**2:** Sauaia A, Moore FA, Moore EE, et al. Epidemiology of trauma deaths: a reassessment. *J Trauma*. 1995;38:185–193.

**3**: MacLeod JBA, Lynn M, McKenney MG, Cohn SM, Murtha M. Early Coagulopathy Predicts Mortality in Trauma. *The Journal of Trauma: Injury, Infection, and Critical Care.* 2003;55(1):39–44.

**4:** Brohi K. Acute Traumatic Coagulopathy. *The Journal of Trauma: Injury, Infection, and Critical Care.* 2003;54(6):1127-1130.

**5:** Frith D, Goslings JC, Gaarder C et al. Definition and drivers of acute traumatic coagulopathy: clinical and experimental investigations. *Journal of Thrombosis and Haemostasis.* 2010;8(9):1919–1925.

**6**: Hess JR, Lindell AL, Stansbury LG, Dutton RP, Scalea TM. The prevalence of abnormal results of conventional coagulation tests on admission to a trauma center. *Transfusion.* 2009;49(1):34–39.

**7:** Eastridge BJ, Malone D, Holcomb JB. Early Predictors of Transfusion and Mortality After Injury: A Review of the Data-Based Literature. *The Journal of Trauma: Injury, Infection, and Critical Care.* 2006;60(Supplement):S20–S25.

**8**: Davenport R, Manson J, De'Ath H, et al. Functional definition and characterization of acute traumatic coagulopathy. *Critical Care Medicine.* 2011;39(12):1-7.

**9:** Spahn DR, Cerny V, Coats TJ, Duranteau J. Management of bleeding following major trauma: a European guideline. *Critical Care* 2007;11(2):414

**10**: Khan S, Davenport R, Raza I, Glasgow S. Damage control resuscitation using blood component therapy in standard doses has a limited effect on coagulopathy during trauma hemorrhage. *Intensive care Medicine* 2015;41(2):239-247.

**11**: Afshari A, Wikkelsø A, Brok J, Møller AM, Wetterlev J.Thrombelastography (TEG) or Thrombelastometry (ROTEM) to Monitor Haemotherapy Versus Usual Care in Patients with Massive Transfusion. *The Cochrane Library* 2011,Issue 3

**12**: Hunt H, Stanworth S, Curry N, Woolley T, Cooper C, Ukoumunne O, Zhelev Z, Hyde C. Thromboelastography (TEG) and rotational thromboelastometry (ROTEM) for trauma induced coagulopathy in adult trauma patients with bleeding. *Cochrane Database Syst Rev.* 2015 Feb 16;2:CD010438.

**13**: Holcomb JB, Tilley BC, Baraniuk S, et al. Transfusion of Plasma, Platelets, and Red Blood Cells in a 1:1:1 vs a 1:1:2 Ratio and Mortality in Patients With Severe Trauma. *JAMA.* 2015;313(5):471–12.

**14:** Johansson et al. Hemostatic resuscitation with plasma and platelets in trauma. *J Emerg Trauma Shock* 2012;5(2):120-5

**15**: Roberts I, Shakur H, Coats T, Hunt B. The CRASH-2 trial: a randomised controlled trial and economic evaluation of the effects of tranexamic acid on death, vascular occlusive events and transfusion requirement in bleeding trauma patients. *Health Technology Assessment* 201;17(10):1-79.

## 12.2 SOFA TABLE OF ORGAN DYSFUNCTION [a]

| SOFA score | 0 | 1 | 2 | 3 | 4 |
|---|---|---|---|---|---|
| **Respirationa** $PaO_2/FIO_2$ (mm Hg) $SaO_2/FIO_2$ | >400 | <400<br>221–301 | <300<br>142–220 | <200<br>67–141 | <100<br><67 |
| **Coagulation** Platelets $10^3/mm^3$ | >150 | <150 | <100 | <50 | <20 |
| **Liver** Bilirubin (mg/dL) | <1.2 | 1.2–1.9 | 2.0–5.9 | 6.0–11.9 | >12.0 |
| **Cardiovascular**[b] Hypotension | No hypotension | MAP <70 | Dopamine </=5 or dobutamine (any) | Dopamine >5 or norepinephrine </=0.1 | Dopamine >15 or norepinephrine >0.1 |
| **CNS** Glasgow Coma Score | 15 | 13–14 | 10–12 | 6–9 | <6 |
| **Renal** Creatinine (mg/dL) or urine output (mL/d) | <1.2 | 1.2–1.9 | 2.0–3.4 | 3.5–4.9 or <500 | >5.0 or <200 |

[a] *Created in a consensus meeting of the European Society of Intensive Care Medicine in 1994 and further revised in 1996.*

[b] *Adrenergic agents administrated for at least 1 hr (doses given are in µg/kg/min)*

## 12.3 LIST OF MANAGEMENT DOCUMENTS

The following is a list of attachments, those with an asterisk* will be submitted to the Research Ethics Committee with the protocol:

- Consent Form*
- Patient Information Sheet*
- Source Data Identification List (see *Section 10.4*)
- Core Lab Instructions To Investigators (see *Section 12.4*)

## 12.4 CORE LAB INSTRUCTIONS TO INVESTIGATORS

The purpose of this section is to describe requirements for study sample collection, processing and analysis.

***Whole blood (20ml)***

Collect 20ml whole blood in appropriate collection vessels:

- 2 x 2.7ml vacutainer (containing Sodium Citrate) *CCT & VHA*
- 1 x 2.0ml arterial blood gas syringe *Arterial blood gas*
- 1 x 4.0ml vacutainer (containing dipotassium EDTA) *Full blood count*
- 1 x 5.0ml vacutainer (containing silica clot activator) *Biochemistry*

# 12.5 DEFINITIONS OF TRANSFUSION REACTIONS

## 12.5.1 Severity of Acute Transfusion Reactions

The Investigator must assess the severity of the event according to the following terms and assessments. The intensity of an event should not be confused with the term “serious” which is a regulatory definition based on subject/event outcome criteria.

| **Severity Grades for Acute Transfusion Reactions** | | | |
|---|---|---|---|
| **Category** | **1=Mild** | **2=Moderate** | **3=Severe** |
| Febrile type reaction | A rise in temperature up to $2^{0}$C with no other signs/symptoms | A rise in temperature of $2^{0}$C or more, and/or rigors, chills, other inflammatory symptoms /signs such as myalgia or nausea which precipitate stopping the transfusion | A rise in temperature of $2^{0}$C or more, and/or rigors, chills, other inflammatory symptoms signs such as myalgia or nausea and/or hypotension which necessitate stopping the transfusion, medical review and/or hospital admission or prolongation of stay |
| Allergic type reaction | Transient flushing, urticaria or rash | Wheeze or angioedema with or without flushing /urticaria/rash but without respiratory compromise or hypotension | Bronchospasm, stridor, angioedema or circulatory problems which require urgent medical intervention and/or, directly result in or prolong hospital stay or anaphylaxis (severe life threatening,generalized or systemic hypersensitivity reaction with rapidly developing airway and/or breathing and/or circulatory problems, usually associated with skin or mucosal changes) |
| Reaction with both allergic and febrile features | Features of mild febrile and mild allergic reactions | Features of both allergic or febrile reactions, at least one of which is in the moderate category | Features of both allergic or febrile reactions, at least one of which is in the severe category |
| Hypotension | | Isolated fall in systolic or diastolic pressure of 30mm or more in the absence of inflammatory, allergic or anaphylactic symptoms; no/minor intervention required | Hypotension leading to shock without allergic or inflammatory symptoms; urgent medical intervention requires |

*These definitions are reproduced from UK SHOT (Serious Hazards of Transfusion)*

## 12.5.2 Non-Acute Transfusion Reactions

| **Category** | **Definition** |
|---|---|
| **TRALI**<br>(Transfusion related acute lung injury) | Acute dyspnoea with hypoxia and bilateral pulmonary infiltrates during or within six hours of transfusion, not due to circulatory overload or other likely cause. |
| **TACO**<br>(Transfusion associated circulatory overload) | Any four of the following occurring within six hours of transfusion:<br>° Acute respiratory distress.<br>° Tachycardia.<br>° Increased blood pressure.<br>° Acute or worsening pulmonary oedema.<br>° Evidence of positive fluid balance. |
| **TTI**<br>(Transfusion transmitted infection) | Include as a TTI if, following investigation, the recipient had evidence of infection post transfusion,<br>and there was no evidence of infection prior to transfusion and no evidence of an alternative source of infection.<br>Plus;<br>**Either** at least one component received by the infected recipient was donated by a donor who had evidence of the same transmissible infection.<br>**Or** at least one component received by the infected recipient was shown to contain the agent of infection. |

These definitions are reproduced from UK SHOT (Serious Hazards of Transfusion).
