# Supplementary material for: iTACTIC – implementing Treatment Algorithms for the Correction of Trauma-Induced Coagulopathy: study protocol for a multicentre, randomised controlled trial
Source: Trials. 2017 Oct 18;18:486. doi: 10.1186/s13063-017-2224-9 (PMC5648415; doi:10.1186/s13063-017-2224-9)
Supplement: Supplementary file 3 — SOFA score. (ZIP 217 kb) [file 13063_2017_2224_MOESM3_ESM.zip › Additional file 3 - iTACTIC Information Sheet - Subject R2.docx]

**The iTACTIC Study**

**Participant Information Sheet**

**Title of Study: *I****mplementing* ***T****reatment* ***A****lgorithms for the* ***C****ure of* ***T****rauma* ***I****nduced* ***C****oagulopathy - A multi-centre, prospective, randomized controlled study to compare outcomes of viscoelastic haemostatic assay (VHA)-guided resuscitation versus optimised conventional coagulation test (CCT) resuscitation support in haemorrhaging trauma patients.*

**Short Title:** iTACTIC **REC Reference Number**:

**Principal Investigator:** Professor Karim Brohi

**Sponsor:** Queen Mary University of London (QMUL)

**Introduction**

We would like to invite you to participate in a clinical research study. Before you decide, you need to understand why the research is being carried out and what it will involve, so please take time to read the following information carefully. Take independent advice and/or talk to others about the study if you wish.

If you decide that you are willing to take part in this clinical study, we will ask you to read and sign a subject declaration form, a copy of which you will be given to keep. We will keep you fully informed during the study so you can let us know if you have any concerns or if you decide to withdraw from the study.

If you decide that you do not wish to take part, it will not affect the standard of care you receive in any way.

# Key Facts about this study

- You recently suffered a severe injury which led to major bleeding.
- Following the injury, the treating doctors decided that you needed to be resuscitated with the transfusion of blood products as part of your care.
- Currently, patients are transfused blood products based upon a Major Haemorrhage Protocol (MHP) in place in each hospital. A MHP aims to replace the different components found within the blood that have been lost and to assist the injured patient in making adequate blood clots to prevent further bleeding. A MHP uses conventional hospital laboratory tests of blood clotting to guide therapy.
- In approximately one quarter of bleeding trauma patients, the ability to form adequate blood clots is lost. This condition is termed Trauma Induced Coagulopathy (TIC).
- Current MHPs fail to detect TIC early or correct it during major bleeding. Patients with TIC have worse outcomes.
- This research project is designed to find out if a rapid, detailed blood clotting test called Viscoelastic Haemostatic Assays (VHA) can be used to identify TIC early and to guide a MHP for that individual patient’s needs.
- The study aims to compare the outcomes of patients treated using the conventional blood transfusion strategy with the outcomes of patients treated using a personalised blood transfusion strategy guided by VHA.
- We are unsure at present whether or not a personalised transfusion strategy guided by VHA is more effective than conventional transfusion strategies.
- You were eligible to participate in this trial when you required transfusion therapy to treat major bleeding. The treating doctor decided to enter you into the study because you were too ill to make a decision yourself about participation.
- We approach you now to ask whether you would be willing to continue to participate in the study.

The title of this study is: ‘**iTACTIC: Implementing Treatment Algorithms for the Cure of Trauma Induced Coagulopathy** - A multi-centre, prospective, randomized controlled study to compare outcomes of viscoelastic haemostatic assay (VHA)-guided resuscitation versus optimised conventional coagulation test (CCT) resuscitation support in haemorrhaging trauma patients.’

In short, the study looks at using VHA tests to guide blood transfusion therapy for the individual with major bleeding and assesses the effect this has on the participant’s outcome.

**What is the purpose of this study?**

Trauma (severe injury) is a leading cause of death and disability worldwide. Almost half of all trauma deaths are due to bleeding and most of these will occur within six hours from injury. Trauma Induced Coagulopathy (TIC) is an inability to make adequate blood clots following severe injury and it occurs in one quarter of trauma patients. TIC may worsen bleeding and is known to be associated with increased blood transfusion requirements and an increased number of deaths. Despite recent advances in the resuscitation and treatment of bleeding trauma patients, we still fail to correct TIC during ongoing bleeding using conventional Major Haemorrhage Protocols (MHP). Viscoelastic Haemostatic Assays (VHA) provide a comprehensive assessment of blood clot formation and blood clot breakdown. In comparison to conventional coagulation (blood clotting) tests performed in a hospital laboratory, VHA can be performed by the patient’s bedside and provide results within minutes.

The main objective is to determine the optimal guidance of transfusion therapy in bleeding trauma patients. One group of patients taking part in this trial will have VHA performed, with their resuscitation treatment tailored to these results. The second group of patients will receive resuscitation treatment according to the hospital’s standard MHP. The effects of the two treatment regimes will be compared, in particular focusing on differences in transfusion requirements and patient outcomes including survival.

**Why are you being approached?**

You have been entered into this research study because you were admitted to the hospital with major ongoing bleeding following your injury and the admitting doctor decided you needed resuscitation with blood products. Now that you have been entered into the trial, we would like to ask you if you are willing to remain in the study.

**Do I have to take part?**

No, participation is completely voluntary. If you decide that you are willing to participate, you will be given this information sheet to keep and be asked to sign a subject declaration form. If you agree to take part, you are still free to change your mind at any time without giving a reason. A decision not to take part will not affect the standard of care you receive.

**What will happen if I agree?**

You have already been randomly allocated to one of two groups of trial participants. One group was resuscitated using VHA to guide blood product transfusion. The other group was resuscitated using the hospital’s standard MHP, which uses conventional hospital laboratory tests of coagulation to guide blood product transfusion. The group you are in was determined randomly using pre-sealed opaque envelopes. There was a 50/50 chance of you being in either of the groups.

During your stay in hospital, we will review your progress each day. We will record information about your treatment, any other illnesses if they occur and how long you stay in the intensive care unit and in hospital overall. Participation in this study will require additional research blood samples to be collected. These will be collected on admission, during the initial blood transfusion and at set time points up to 24 hours following the injury. The set time points for blood sampling are at 6 and 24 hours after the injury. No research blood samples will be taken after the 24 hour sample or following hospital discharge. The admission and all subsequent research blood samples will involve taking a maximum of 20mls (equivalent to 4 teaspoons) of blood. Once you leave the hospital, or on the 28^th^ day from your injury, whichever comes sooner, we will ask you to complete a questionnaire. The questionnaire will ask how you are feeling and will take about 2 minutes to complete. You will be asked to complete a similar questionnaire again, 3-months after your injury. There is no need to make any special visits to hospital as part of this study.

**What if I do not agree to participate?**

If you do not wish to remain in the trial, the treatment you receive will be according to current standard practice. This trial involves only one extra measure – the use of VHA to guide resuscitation therapy during the initial bleeding episode.

**What is the procedure that is being tested?**

We are testing whether it is possible to improve outcomes for bleeding trauma patients by tailoring their resuscitation with blood products guided by VHA, to provide a personalised, transfusion therapy for that individual patient’s needs.

**What are the possible disadvantages and risks of taking part?**

All patients in this study will have the same access to blood products available as part of a standard MHP. The MHP used in both groups is based upon current best evidence. All the blood products that will be used in the iTACTIC study have been approved for use in the UK and have been treated for viruses and other pathogens according to normal procedures. The group of patients receiving therapy based on conventional laboratory tests of coagulation, will not experience any extra risk compared to standard treatment. As far as we know, there are no additional risks associated with participating in this trial.

**What are the possible benefits of taking part?**

There is no known direct benefit from participation in this study at the present time. Participants receiving VHA guided resuscitation might have better outcomes, but at present we do not know if this will be the case. The outcome of this trial will help to determine any benefits associated with the use of VHA for bleeding trauma patients.

**What if there is a problem?**

If you have any concern about any aspect of this study, you should ask to speak to the researchers who will do their best to answer any questions. If you remain unhappy and wish to complain formally, you can do this through the usual hospital procedures.

**Will my personal information be kept confidential if I participate in this study?**

All information which is collected about you during the course of the research will be kept strictly confidential and will be stored securely in coded form. Data collected may be sent outside of the Trust, to other participating European trauma research centres for statistical analysis, but un-coded identifiable personal patient information will not leave the local research group. If you consent to take part in this study, the people conducting the study will abide by the Data Protection Act 1998, and the rights you have under this Act. Only authorised personnel such as researchers and research auditors will have access to the information. We will store your information for 20 years until the end of this research project.

**What if new information becomes available?**

Sometimes during the course of a research project, new information becomes available about the treatment that is being studied. If this happens, the research team will tell you about it and discuss further participation in the study.

**What will happen if I don’t want to carry on with the study?**

If you decide to stop participation in this study, recorded information already collected from your medical records may still be used for the study. We will ask if we can still use this data we have collected so far, but if you disagreed we would remove it from the study database. You will continue to be monitored for safety.

**What will happen to the results of the study?**

Once the study is completed the results will be published in Scientific and Medical Journals and presented at meetings of health professionals. You will not be identifiable in any publications or presentations resulting from this study.

**What will happen to my blood samples taken?**

All blood samples collected from you during this study will be destroyed according to local hospital procedures once testing on them has been completed. There will be no further storage or testing of the samples.

**Will any genetic tests be done?**

No genetic tests will be done on the samples collected as part of this study.

**Who has reviewed the study?**

All research in the NHS is looked at by an independent group of people, called the Research Ethics Committee in order to protect patient safety, rights, wellbeing and dignity. This study has been reviewed and given favourable opinion by the National Research Ethics Service (NRES) Committee London – City & East.

**Who should I contact for further information?**

If at any time during the study you have questions or concerns regarding the study you can contact the local Principal Investigator, who is in charge of the research at your hospital:

Professor Karim Brohi, Principal Investigator (via secretary) on 0203 594 0723 or Miss Claire Rourke, Trial Manager on 0203 594 0731.

If you require impartial, local advice, please contact the Patient Advice and Liaison Service, telephone: 020 359 42040/42050 or e-mail: pals@bartshealth.nhs.uk

**Thank you for taking the time to consider your continued participation in the iTACTIC study.**
