# Supplementary material for: iTACTIC – implementing Treatment Algorithms for the Correction of Trauma-Induced Coagulopathy: study protocol for a multicentre, randomised controlled trial
Source: Trials. 2017 Oct 18;18:486. doi: 10.1186/s13063-017-2224-9 (PMC5648415; doi:10.1186/s13063-017-2224-9)
Supplement: Supplementary file 3 — SOFA score. (ZIP 217 kb) [file 13063_2017_2224_MOESM3_ESM.zip › Additional file 3 - SOFA scoreR2.pdf]

SOFA score	0	1	2	3	4
**Respirationa** PaO_2/FIO_2 (mm Hg) SaO_2/FIO_2	>400	<400 221–301	<300 142–220	<200 67–141	<100 <67
**Coagulation** Platelets $10^3/mm^3$	>150	<150	<100	<50	<20
**Liver** Bilirubin (mg/dL)	<1.2	1.2–1.9	2.0–5.9	6.0–11.9	>12.0
**Cardiovascular**[b] Hypotension	No hypotension	MAP <70	Dopamine </=5 or dobutamine (any)	Dopamine >5 or norepinephrine </=0.1	Dopamine >15 or norepinephrine >0.1
**CNS** Glasgow Coma Score	15	13–14	10–12	6–9	<6
**Renal** Creatinine (mg/dL) or urine output (mL/d)	<1.2	1.2–1.9	2.0–3.4	3.5–4.9 or <500	>5.0 or <200

[a] *Created in a consensus meeting of the European Society of Intensive Care Medicine in 1994 and further revised in 1996.*

[b] *Adrenergic agents administrated for at least 1 hr (doses given are in μg/kg/min)*
